# FXR deficiency induced ferroptosis via modulation of the CBP-dependent p53 acetylation to suppress breast cancer growth and metastasis

**DOI:** 10.1038/s41419-024-07222-3

**Published:** 2024-11-14

**Authors:** Ping Huang, Han Zhao, Hua Dai, Jinying Li, Xiafang Pan, Wentian Pan, Chunhua Xia, Fanglan Liu

**Affiliations:** 1 School of Pharmacy, Jiangxi Medical College, Nanchang University, Nanchang, 330031 P. R. China; 2 Department of Pathology, the First Affiliated Hospital of Nanchang University, Nanchang, 330038 P. R. China; 3 Jiangxi Province Key Laboratory of New Drug Evaluation and Transformation, Nanchang, 330031 P. R. China

**Keywords:** Breast cancer, Prognostic markers

## Abstract

Farnesoid X receptor (NR1H4/FXR) functions as a scavenger of lipid peroxide products and drives the proliferation and metastasis of various cancers. However, the underlying molecular mechanisms remain poorly understood. In our study, we found that the expression levels of FXR, vimentin and SLC7A11 were significantly higher in breast cancer tissues, particularly in metastatic cancer tissues compared to non-metastatic ones. Furthermore, the increased FXR expression was positively correlated with vimentin and SLC7A11 in clinical tumor specimens. In addition, a high level of FXR correlated with poor prognosis in patients with breast cancer. Both Z-Guggulsterone (Z-GS), as a pharmacological inhibitor of FXR, and silencing FXR curbed proliferation and migration of breast cancer cells by promoting ferroptosis. Notably, our results showed that FXR competitively bound to CREB-binding protein (CBP) to suppress the interaction between p53 and CBP in the nucleus, and thus prevented p53 acetylation at lys382, which was essential for upregulating the expression of SLC7A11. Conversely, FXR knockdown increased the interaction between p53 and CBP and promoted p53 acetylation, which ultimately led to facilitating ferroptosis in breast cancer cells. More importantly, we also found that Z-GS inhibited TGF-β1-induced tumor growth and metastasis of breast cancer primarily through ferroptosis via regulating CBP-dependent p53 acetylation in nude mice. In conclusion, the FXR was first reported as a tumor promoter that enhanced the proliferation and metastasis of breast cancer cells through regulating CBP-dependent p53 K382 acetylation. It proposes that FXR may serve as a potential therapeutic target for the treatment of breast cancer.

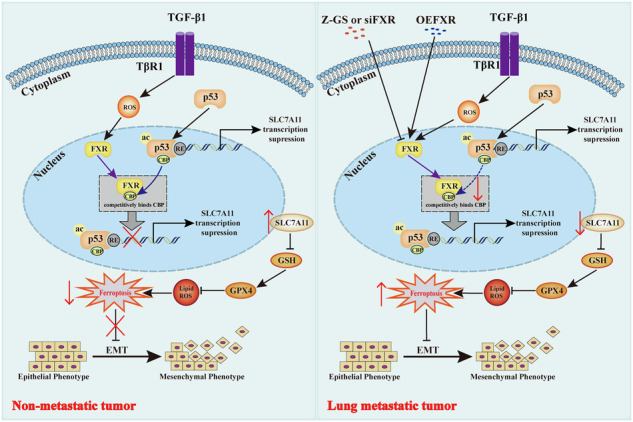

## Introduction

Breast cancer is a prevalent malignant tumor among women worldwide, with a steadily increasing incidence rate. It ranks as one of the most common tumors globally [1], posing a significant threat to public health. Presently, breast cancer treatment primarily consists of surgery, postoperative radiotherapy, and chemotherapy. However, these treatments can lead to adverse reactions and complications, diminishing patients’ quality of life [2, 3]. Additionally, tumor recurrence and metastasis remain formidable challenges in breast cancer treatment [4]. Therefore, understanding the mechanisms underlying tumor metastasis is crucial for improving postoperative survival rates and enhancing the quality of life for breast cancer patients.

Farnesoid X receptor (NR1H4/FXR), functioning as a bile acid receptor (BAR), is also a nuclear receptor implicated in various cellular processes in cancer [5]. Previous research has indicated that FXR exhibited tissue-specific and cancer-specific functions, served as an oncoprotein or a tumor suppressor in tumor progression. On the one hand, FXR activation hindered the expression of epithelial-mesenchymal transition (EMT) markers, such as slug, vimentin, snail, fibronectin, and MMP-9, thereby restraining the migration of colon cancer cells [6]. On the other hand, FXR activation fostered the EMT process in hepatocellular carcinoma by regulating N-Cadherin and phosphorylated focal adhesion kinase (p-FAK) expression [7]. Elevated FXR expression is associated with invasion and metastasis of breast cancer, leading to reduced survival rates for breast cancer patients [8]. In addition, activation of FXR has been shown to promote bone metastasis in breast cancer cells, whereas treatment with the FXR antagonist Z-Guggulsterone (Z-GS) impedes migration by induction of apoptosis [9]. However, the detailed functional role of FXR in breast cancer and the underlying mechanisms remain unknown.

Ferroptosis represents a novel form of cell death distinct from necrosis, apoptosis, and autophagy, which is characterized by the uncontrolled accumulation of lipid ROS and increased iron levels [10]. Ferroptosis plays a crucial role in various diseases, including cancer, inflammation, and cardiovascular diseases [11–13]. Increasing evidence has revealed that lipids and iron metabolism pathways are closely associated with cancer progression [14], particularly cancer cells in the mesenchymal state are prone to metastasis and highly susceptible to ferroptosis [15, 16]. Solute carrier family 7 member 11 (SLC7A11), a subunit of the Xc- system, acts as a specific amino acid transporter and a critical regulator of ferroptosis [17, 18]. SLC7A11 deficiency reduces cystine uptake, resulting in cystine-dependent glutathione peroxidase inactivation, accompanied with increasing intracellular lipid peroxidation and induction of ferroptosis [19, 20]. A newly emerging mechanism underlies that SLC7A11 is a key target of p53, as it inhibits cystine absorption via down-regulation of SLC7A11 expression, leading to subsequent cell ferroptosis [21–23]. Acetylation of p53 within its DNA binding domain inhibits SLC7A11 expression, while an acetylation-defective mutant p53, with a specific lysine-to-arginine mutation, restores SLC7A11 expression and inhibits ferroptosis [24]. Moreover, Yang et al. has shown that STAT6 competitively binds to CREB-binding protein (CBP), thereby inhibiting the interaction between p53 and CBP, reducing p53 acetylation, and negatively modulating induction of ferroptosis [25]. There is increasing evidence that FXR plays an important role in ferroptosis by regulating cellular lipid metabolism and iron homeostasis ^26^. For instance, FXR regulates the transcription of lipid metabolism-related genes and mitigates cisplatin-induced acute kidney injury [26]. Nonetheless, the mechanism by which FXR regulates ferroptosis in breast cancer metastasis remains elusive.

Here, we first determined that FXR was upregulated in breast cancer cells undergoing EMT during carcinogenesis, which was correlated with a poor prognosis in breast cancer patients. Both in breast cancer cells and mice models, FXR deficiency (Z-Guggulsterone(Z-GS) or siRNA) curbed cell proliferation and migration by promoting ferroptosis. Mechanistically, our results showed that FXR knockdown by siRNA increased the binding of CBP to p53, promoting p53 K382 acetylation and facilitating ferroptosis. Taken together, our findings not only emphasize the crucial role of FXR in breast cancer metastasis but also investigate its novel regulatory mechanism of ferroptosis through modulation of the CBP-dependent p53 acetylation, which suggest that FXR may function as a potential therapeutic target in metastatic breast cancer.

## Results

### Increasing expression of SLC7A11 is correlated with vimentin in breast cancer patients

**Fig. 1:**
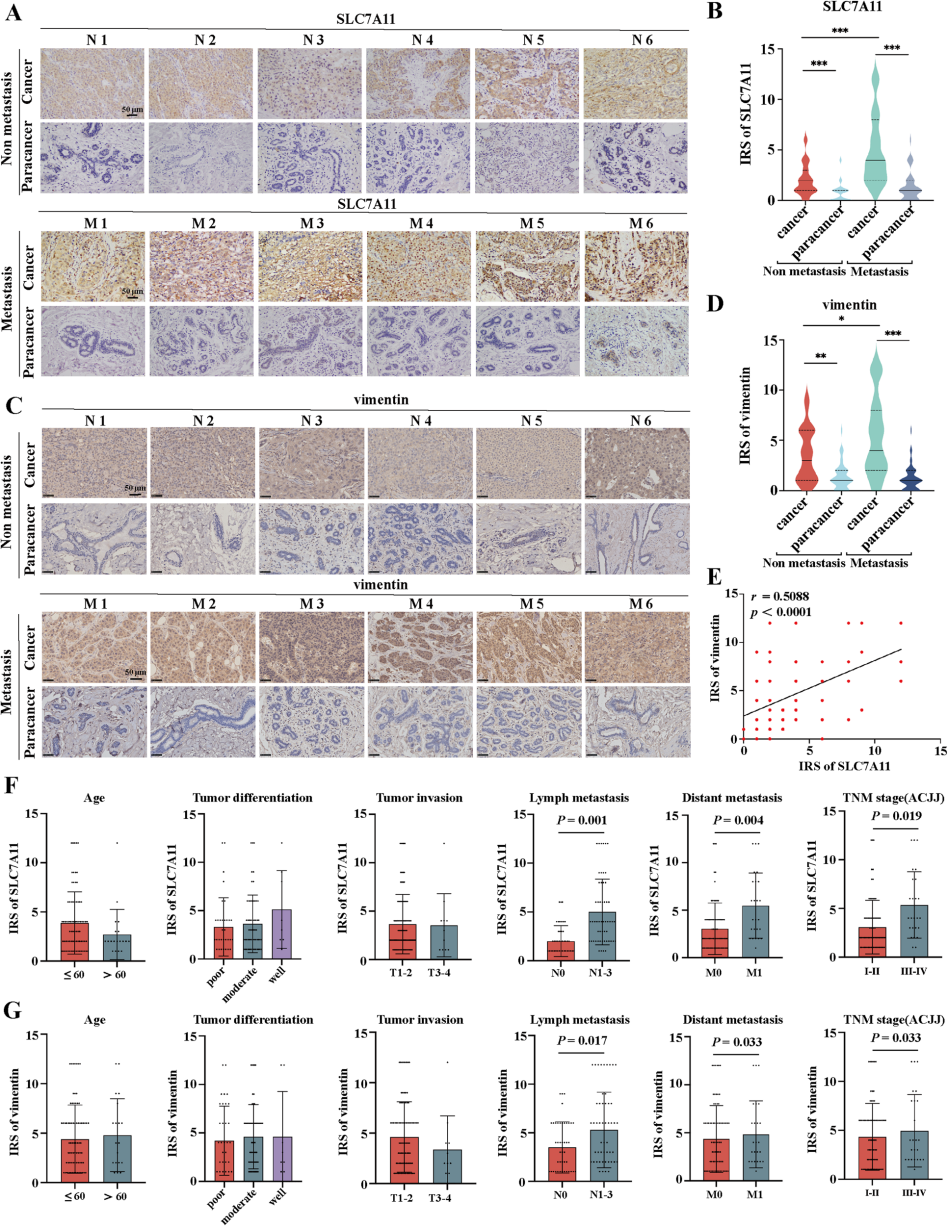
Increasing expression of SLC7A11 is correlated with vimentin in breast cancer patients. **A**, **C** Immunohistochemistry analysis of SLC7A11 and vimentin were performed in breast cancer tissues and adjacent tissues with and without metastasis. **B**, **D** Immunoreactive scores (IRSs) of SLC7A11 and vimentin were calculated. **E** Correlation and simple linear regression of vimentin and SLC7A11 in 101 clinical breast cancer tissues. **F**, **G** The IRSs of SLC7A11 and vimentin were compared within the following groups: age, tumor differentiation, tumor invasion, lymph metastasis, distant metastasis and the TNM stage. Scale bar: 50 µm. N, non metastasis. M, metastasis. The data are expressed as the mean ± SD (n = 101); ^*^p ≤ 0.05, ^**^p ≤ 0.01, and ^***^p ≤ 0.001.

SLC7A11, served as a key target for regulating ferroptosis, plays a crucial role in maintaining redox homeostasis and defending against lipid peroxidation injury during tumor progression [27]. To investigate the relationship between SLC7A11 expression and EMT progress during carcinogenesis, we performed immunohistochemistry in collected paraffin sections of tumors of 101 patients with breast cancer and matched adjacent normal tissues (Fig. 1A–D). The expression levels of SLC7A11 and vimentin were higher in cancer tissues compared with normal tissues, especially the IRSs of SLC7A11 and vimentin were higher in metastatic cancer tissues than in non-metastatic cancer tissues. Breast cancer patients with lymph node metastasis, distant metastasis or late TNM stage luminal-like subtype (stage III&IV), have higher levels of SLC7A11 and vimentin (Fig. 1F, G). Furthermore, The expression level of SLC7A11 has a significant correlation with the expression of vimentin in breast cancer (*r* = 0.5088, *p* < 0.0001) (Fig. 1E). These results suggested that SLC7A11 accumulation may be triggered by EMT process, which was associated with the metastasis of breast cancer.

### Accumulation of FXR is associated with poor prognosis in breast cancer patients

**Fig. 2:**
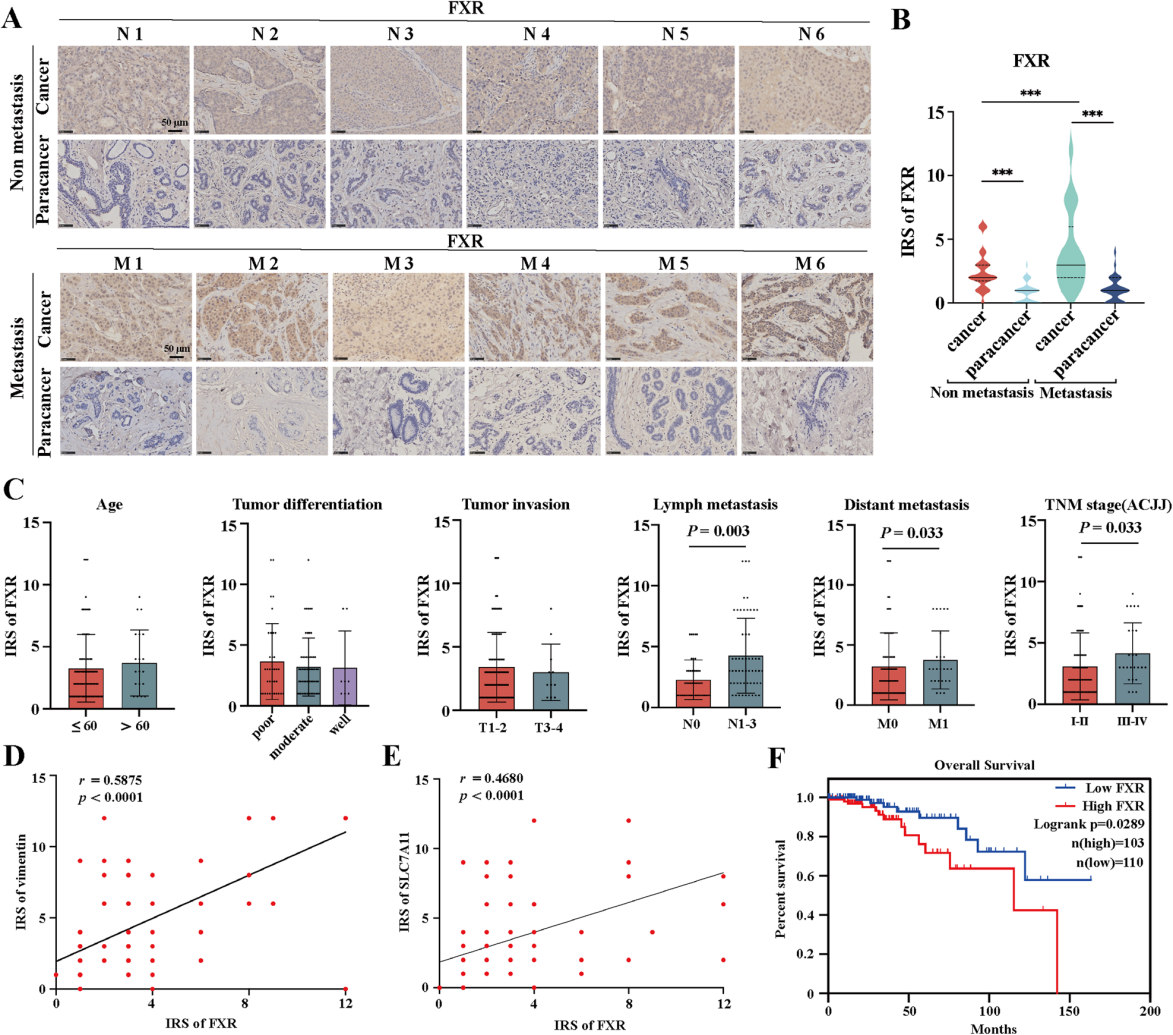
Accumulation of FXR is associated with poor prognosis in breast cancer patients. **A** Immunohistochemistry analysis of FXR was performed in breast cancer tissues and adjacent tissues with and without metastasis. **B** Immunoreactive score (IRS) of FXR was calculated. **C** The IRS of FXR was compared by age, tumor differentiation, tumor invasion, lymph metastasis, distant metastasis and the TNM stage. **D**, **E** Correlation and simple linear regression of FXR and vimentin or SLC7A11 levels in 101 clinical breast cancer tissues. **F** Kaplan-Meir survival analysis of the expression of FXR in breast cancer patients was performed using TCGA. Scale bar: 50 µm. N, non metastasis. M, metastasis. The data are expressed as the mean ± SD (n = 101); ^*^p ≤ 0.05, ^**^p ≤ 0.01, and ^***^p ≤ 0.001.

It has been reported that high expression of FXR is associated with invasion and metastasis of breast cancer, leading to reduced survival rates in breast cancer patients [8]. To investigate the critical role of FXR in breast cancer metastasis, immunohistochemistry analysis was performed and the results showed that the immunoreactive score (IRS) of FXR was significantly higher in cancer tissues than in normal tissues, especially in metastatic cancer tissues compared to non-metastatic ones, which indicated that high expression of FXR was correlated to the metastasis of breast cancer (Fig. 2A, B). Besides, our results indicated that the expression of FXR was positively associated with lymph node metastasis, distant metastasis and late TNM stage luminal-like subtype (stage III&IV), but no statistical difference in FXR expression was observed based on age, tumor differentiation and tumor invasion (Fig. 2C). Increased FXR expression was positively correlated with vimentin levels (*r* = 0.5875, *p* < 0.0001) and SLC7A11 levels (*r* = 0.4680, *p* < 0.0001) (Fig. 2D, E). Moreover, TCGA sequencing data indicated a link between high FXR mRNA levels and poor overall survival (logrank *p* = 0.0289, Fig. 2F). Above all, these results revealed that FXR was upregulated in breast cancer cells undergoing EMT during carcinogenesis, which was correlated to a poor prognosis in breast cancer. FXR deficiency may be a key factor for breast cancer cells to induce ferroptosis by regulating SLC7A11 expressions.

### FXR antagonist Z-GS inhibits cell proliferation and migration mainly by inducing ferroptosis in breast cancer cells

**Fig. 3:**
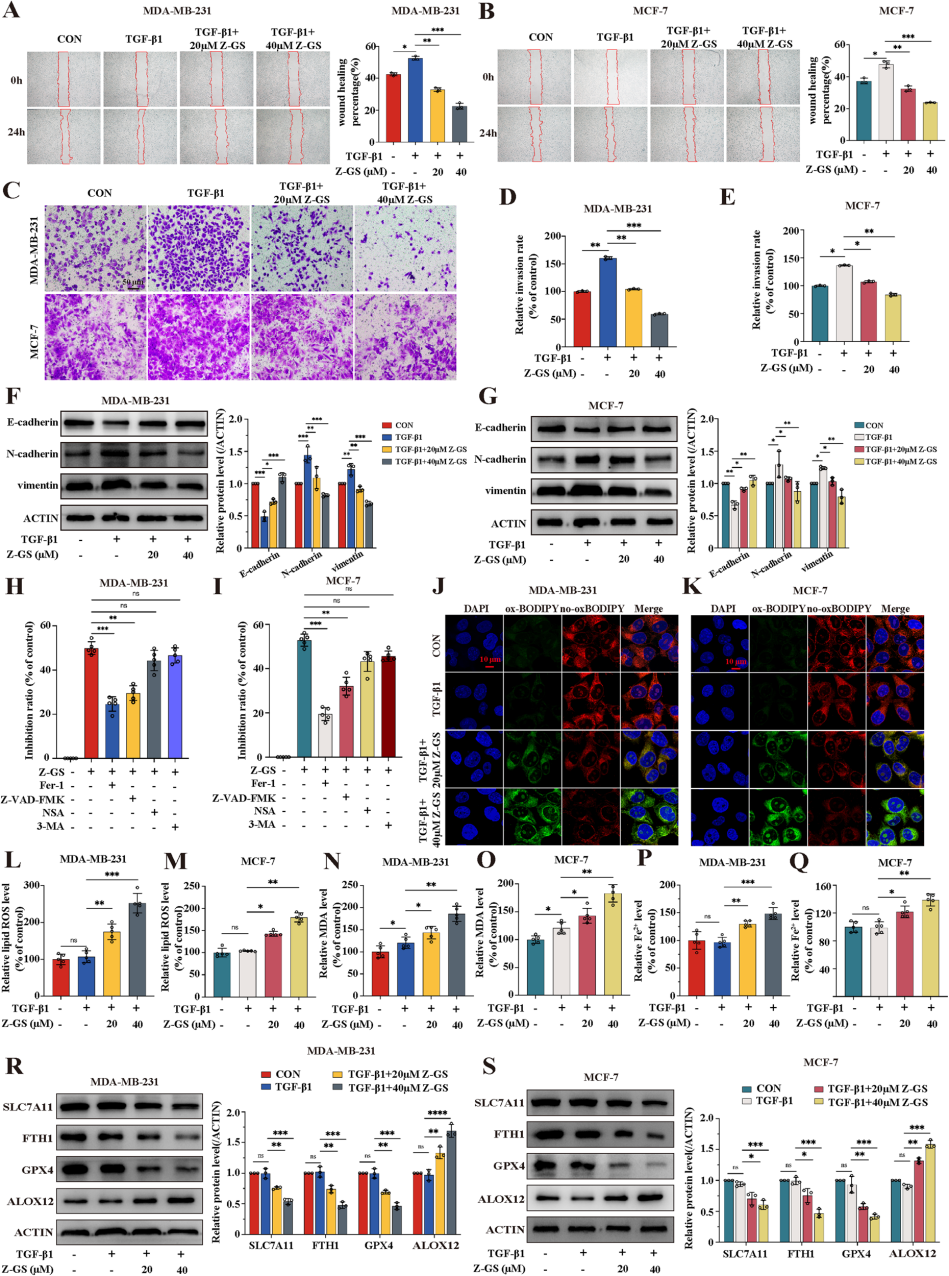
FXR antagonist Z-GS inhibits cell proliferation and migration mainly by inducing ferroptosis in breast cancer cells. **A**–**E** Wound healing assay, transwell invasion assay were performed to evaluate the migration and invasion ability of breast cancer cells; n = 3 samples. **F**, **G** The proteins levels of E-Cadherin, N-Cadherin, and vimentin in MDA-MB-231 and MCF-7 cells were measured by western blotting assays; n = 3 samples. **H**, **I** The viability of MDA-MB-231 and MCF-7 cells cultured with Z-GS in the presence or absence of various cell death inhibitors was detected by CCK8; n = 5 samples. **J**–**M** The relative levels of lipid ROS were detected by the C11-BODIPY probe in MDA-MB-231 and MCF-7 cells; all images were captured using a confocal microscope; n = 5 samples. **N**–**Q** Cellular MDA and Fe^2+^ levels were evaluated using commercial kits; n = 5 samples. **R**, **S** The expression levels of SLC7A11, FTH1, GPX4 and ALOX12 were examined by western blotting assays; n = 3 samples. Cont., control; TGF-β1, 10 ng/mL; Z-GS, 20 and 40 µM; Fer-1, 1 µmol/L; Z-VAD-FMK, 40 µmol/L; NSA, 2.5 µmol/L; 3-MA, 1 mmol/L. The data are expressed as the mean ± SD; ^*^p ≤ 0.05, ^**^p ≤ 0.01, and ^***^p ≤ 0.001.

To determine the biological function of FXR in breast cancer cells, we employed the FXR antagonist Z-GS and observed its cytotoxic and inhibitory effects on MCF-10A, MDA-MB-231, and MCF-7 cells. In CCK8 assays, Z-GS displayed an inhibitory effect on the proliferation of MDA-MB-231 and MCF-7 cells, while it had no significant impact on MCF-10A cells, which was also confirmed by colony formation assay (Fig. S1). Besides, the results of wound-healing assays and transwell assays showed that 10 ng/mL TGF-β1 significantly increased the invasion and migration of breast cancer cells compared to controls, while Z-GS treatment significantly reduced the migration and invasion abilities of breast cancer cells pretreated with TGF-β1 (Fig. 3A–E). Additionally, western blotting results demonstrated that Z-GS suppressed EMT in breast cancer cells by upregulating the epithelial markers E-Cadherin and downregulating the mesenchymal markers N-cadherin and vimentin (Fig. 3F, G). It’s worth noting that autophagy inhibitor 3-methyladenine (3-MA) and necrosis inhibitor necrosulfonamide (NSA) did not protect against Z-GS-induced cell death in breast cancer cells, while the ferroptosis inhibitor Ferrostatin-1 (Fer-1) and apoptosis inhibitor Z-VAD-FMK significantly reversed Z-GS-induced cell death, with Fer-1 having a more pronounced effect than Z-VAD-FMK (Fig. 3H, I). To further confirm ferroptosis was indeed induced by Z-GS, we assessed intracellular lipid ROS, MDA, and Fe^2+^ levels in breast cancer cells. As shown in Fig. 3J–Q, Z-GS treatment significantly increased lipid ROS, MDA, and Fe^2+^ levels, particularly with 40 µM Z-GS. Furthermore, Z-GS markedly suppressed the expression of negative regulatory proteins of ferroptosis, including SLC7A11, FTH1, and GPX4 and increased the expression of ferroptosis-related protein ALOX12 (Fig. 3R, S). In addition, TGF-β1 treatment significantly facilitated the accumulation of ROS, accompanied with a compensatory increase of FXR in the anti-oxidative stress response, while Z-GS inhibited the expression of FXR, leading to elevated levels of ROS (Fig. S2). In summary, the FXR antagonist Z-GS inhibited the invasion and migration of breast cancer cells mainly by ferroptosis activation.

### siFXR-mediated ferroptosis inhibits TGF-β1-induced invasion and migration of breast cancer cells

**Fig. 4:**
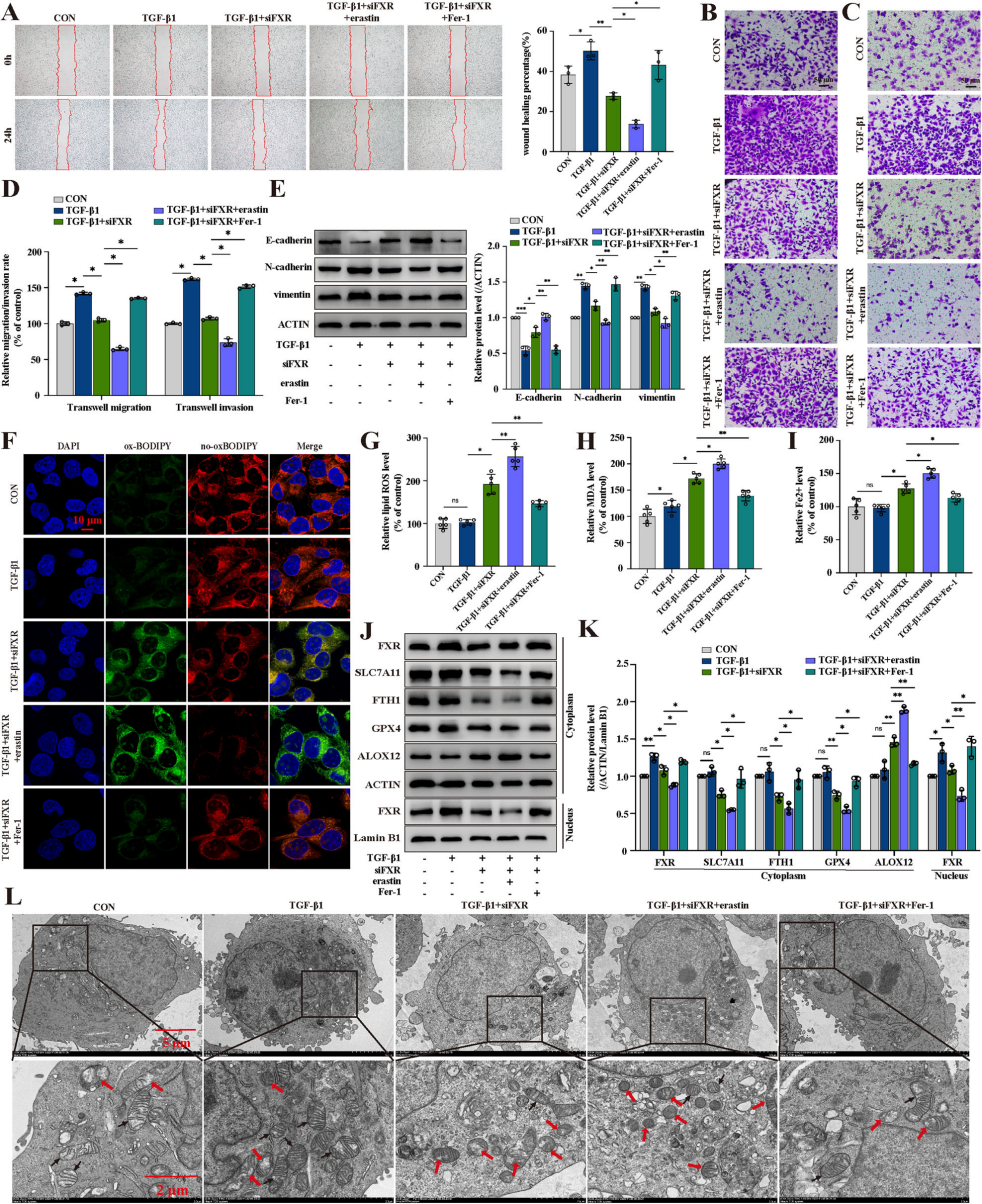
siFXR-mediated ferroptosis inhibits TGF-β1-induced invasion and migration of breast cancer cells. **A**–**D** Wound healing assay, transwell migration assay, and transwell invasion assay were performed to assess the migration and invasion ability of MDA-MB-231 cells; n = 3 samples. **E** The expression levels of E-Cadherin, N-Cadherin, and vimentin were detected by western blotting assays; n = 3 samples. **F**, **G** The relative levels of lipid ROS were detected by the C11-BODIPY probe, and the images were captured using a fluorescence microscope; n = 5 samples. **H**, **I** Cellular MDA and Fe^2+^ levels were evaluated using commercial kits; n = 5 samples. **J, K** The expression levels of FXR, SLC7A11, FTH1, GPX4 and ALOX12 in the cytoplasm and the expression of FXR in the nucleus were detected by western blotting assays; n = 3 samples. **L** The ultrastructure of ferroptosis was detected by TEM; red arrows indicate decreased cristal membrane density in the mitochondria, and black arrows indicate normal mitochondria; n = 3 samples. Cont., control; TGF-β1, 10 ng/mL; erastin, 5 µM; Fer-1, 1 µM. The data are expressed as the mean ± SD; ^*^p ≤ 0.05, ^**^p ≤ 0.01, and ^***^p ≤ 0.001.

To further investigate the role of FXR-regulated ferroptosis in breast cancer cell metastasis, we performed siFXR transfection in MCF-7 cells (Fig. S3A–C). As expected, siFXR significantly inhibited the invasion and migration of breast cancer cells through wound-healing assays and transwell assays. These effects were enhanced by co-treatment with the ferroptosis inducer erastin and reversed by co-treatment with the ferroptosis inhibitor Fer-1 (Fig. 4A–D). Western blotting analysis confirmed that siFXR reversed TGF-β1-induced metastasis of breast cancer cells by downregulating mesenchymal markers (N-cadherin and vimentin) and upregulating the epithelial marker E-Cadherin. These changes were significantly enhanced by erastin and effectively weakened by Fer-1 (Fig. 4E), indicating that siFXR suppressed TGF-β1-induced metastasis of breast cancer cells by inhibiting the EMT process. Then, we assessed several ferroptosis indicators in MDA-MB-231 cells, including lipid ROS levels, MDA levels, and Fe^2+^ levels. The results showed that lipid ROS, MDA, and Fe^2+^ levels were significantly increased after treatment with siFXR. These changes were significantly enhanced by erastin and largely reversed by Fer-1 (Fig. 4F–I). Conversely, FXR overexpression decreased lipid ROS levels and promoted TGF-β1-induced migration of breast cancer cells (Fig. S4). In addition, TEM was performed to observe the ultrastructure of mitochondria and confirm the occurrence of ferroptosis. As shown in Fig. 4L, siFXR-treated cells showed shrinkage of mitochondria and mitochondrial membrane rupture, accompanied by an increase in membrane density, and ridge reduction or even disappearance. These changes were significantly attenuated after treatment with Fer-1. Furthermore, western blotting analysis revealed that negative regulatory proteins of ferroptosis (SLC7A11, GPX4, and FTH1) were downregulated and ferroptosis-related protein ALOX12 was upregulated by siFXR treatment, while these effects were strongly reversed by Fer-1 (Fig. 4J, K). In summary, siFXR-induced ferroptosis effectively inhibited TGF-β1-induced invasion and migration in breast cancer cells.

### FXR competitively binds with CBP and attenuates p53 binding to the promoter of SLC7A11 in breast cancer cells

**Fig. 5:**
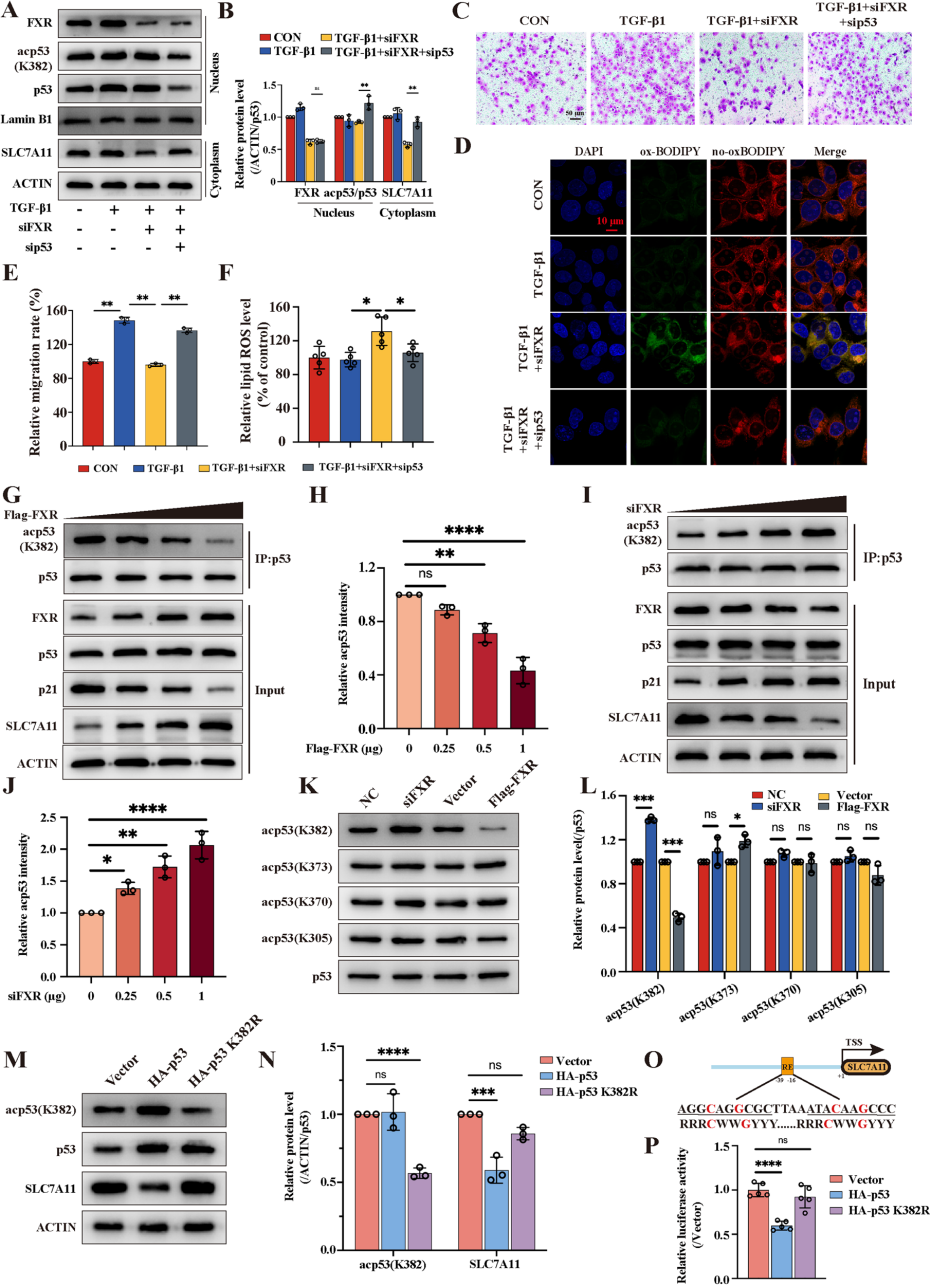
FXR regulated p53 K382 acetylation is responsible for induction of ferroptosis. **A**, **B** The expression levels of FXR, acetyl-p53 (K382), p53 in the nucleus and SLC7A11 in the cytoplasm were assessed by western blotting assays; n = 3 samples. **C** Transwell migration assay was performed to evaluate the migration ability of MDA-MB-231 transfected with sip53; n = 3 samples. **D** The levels of lipid ROS were evaluated by the C11-BODIPY probe in each group, and the images were obtained using a fluorescence microscope; n = 5 samples. **E**, **F** The quantification of transwell migration assays and the relative levels of lipid ROS were evaluated. **G**–**J** MDA-MB-231 cells were transfected with 0, 0.25, 0.5, 1 μg Flag-FXR or 0, 0.25, 0.5, 1 μg siFXR and harvested for CoIP with an anti-p53 antibody; n = 3 samples. **K**, **L** The expression levels of p53, acp53(K382), acp53(K373), acp53(K370) and acp53(K305) were detected and the quantification was calculated; n = 3 samples. **M**, **N** The expression of p53, acp53(K382) and SLC7A11 were detected and the quantification were calculated; n = 3 samples. **O** A schematic diagram of the p53 binding site and sequence on the human SLC7A11 gene (R, A/G; W, A/T; Y, C/T; red indicates the crucial parts for binding p53); TSS: transcription start site. **P** The luciferase activity was detected and analyzed in HEK293T co-transfected with vector or p53 or p53 K382R plasmid and SLC7A11 promoter plasmid; n = 5 samples. The data are expressed as the mean ± SD; *p ≤ 0.05, **p ≤ 0.01, and ***p ≤ 0.001.

p53 is recognized as a tumor suppressor gene that has been reported to undergo acetylation and regulate multiple downstream target genes, such as altering the activity of transcription factor SLC7A11 [23]. To investigate whether p53 was involved in the regulation of SLC7A11 expression in siFXR-induced ferroptosis, we performed p53-siRNA transfection (Fig. S3D). Blocking the p53 pathway with siRNA reversed the siFXR-induced suppression of SLC7A11 expression in TGF-β1-treated cells (Fig. 5A, B). Moreover, the migration ability and lipid ROS assays confirmed that FXR deficiency induced ferroptosis to suppress tumor progression in an p53-dependent manner (Fig. 5C–F). To further explore the potential regulation of FXR on p53 signaling, we overexpressed FXR using a Flag-FXR plasmid and inhibited FXR expression induced by siRNA in MDA-MB-231 cells (Fig. S3E–G). CoIP analysis revealed that the expression of p21, as well as acetyl-p53, but not p53 itself, were dose-dependently downregulated by FXR overexpression, while this effect was dramatically reversed by FXR knockdown (Fig. 5G–J). Additionally, western blotting analysis demonstrated that FXR overexpression markedly increased p53 K382 acetylation, but not the other three lysine residues (K373, K370, K305) in the C-terminal domain of p53, while silencing FXR can mitigate this effect (Fig. 5K, L). The results of western blotting and dual-luciferase reporter assay further revealed that the p53 K382R mutant almost led to abolishing the suppressive effect on SLC7A11 transcriptional activity (Fig. 5M–P). Overall, these observations suggest that FXR regulated p53 K382 acetylation is responsible for induction of ferroptosis in MDA-MB-231 cells.

**Fig. 6:**
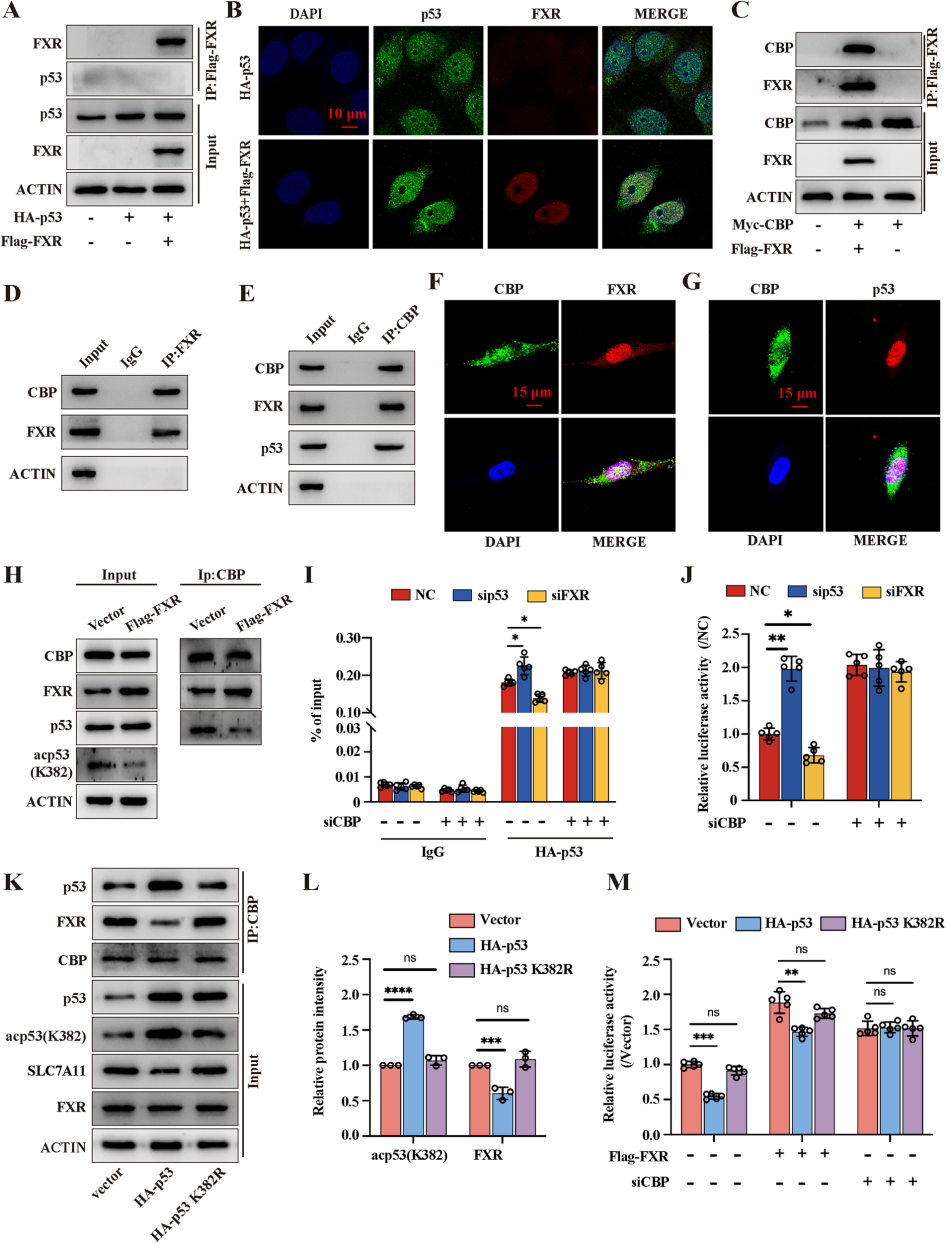
FXR competitively binds with CBP and attenuates the inhibition of p53 on SLC7A11 expression in breast cancer cells. **A** The location of FXR and p53 was determined by immunocoprecipitation assay; n = 3 samples. **B** Cellular interaction between FXR and p53 was determined by immunofluorescence assay in MDA-MB-231 cells; n = 3 samples. **C**–**E** Cellular interaction between FXR and CBP was determined by immunocoprecipitation assay in MDA-MB-231 cells; n = 3 samples. **F** The immunofluorescence assay was used to detect the location of FXR and CBP; n = 3 samples. **G** The immunofluorescence assay was used to detect the location of p53 and CBP; n = 3 samples. **H** Endogenous CBP in control or FXR-overexpression cells was immunprecipicated with an anti-CBP antibody, and the samples were analyzed by western blotting; n = 3 samples. **I** ChIP assay was performed to determine the suppressive effect of p53 on the promoter of SLC7A11; n = 5 samples. **J** The luciferase activity was detected and analyzed in HEK293T co-transfected with given plasmid and SLC7A11 promoter plasmid; n = 5 samples. **K**, **L** MDA-MB-231 cells were transfected with vector or p53 or p53 K382R plasmid and harvested for immunocoprecipitation assay with an anti-CBP antibody, and the samples were analyzed by western blotting; n = 3 samples. **M** The luciferase activity was detected and analyzed in HEK293T co-transfected with given plasmid and SLC7A11 promoter plasmid; n = 5 samples. The data are expressed as the mean ± SD; *p ≤ 0.05, **p ≤ 0.01, and ***p ≤ 0.001.

p53 acetylation regulates the transcription of downstream target genes by interacting with its response elements (RE), and CBP is a key acetyltransferase involved in p53 acetylation and contributes to regulate the stability and transactivation of p53 [28]. To further validate that CBP is involved in FXR deficiency induced p53 acetylation, we performed CoIP, confocal microscopy experiments, dual-luciferase reporter and ChIP assays. The results of CoIP and confocal microscopy experiments showed that there was no direct binding between FXR and p53, and the location of p53 was not affected by FXR (Fig. 6A, B). Subsequently, the results confirmed that FXR interacts with CBP (Fig. 6C, D). In addition, we also found that a direct interaction between CBP and FXR, p53 and CBP (Fig. 6E–G). Notably, p53 acetylation was supressed after FXR overexpression, and we speculated that FXR and p53 may compete for CBP binding. Indeed, the interaction between CBP and p53 was significantly inhibited in FXR-overexpressing cells (Fig. 6H and Fig. S5). Moreover, knockdown of CBP inhibited siFXR-induced p53 acetylation and led to a significant increase in the expression of SLC7A11 (Fig. 6I, J). Furthermore, the CoIP assays demonstrated that p53 mutant with amino acid substitution K382 abolished its interaction with CBP. Interestingly, the mutation also led to an increase in the interaction between FXR and CBP (Fig. 6K, L). Additionally, the dual-luciferase reporter confirmed that FXR overexpression or CBP knockdown attenuated the suppressive effect on SLC7A11 expression. Meanwhile, the mutation in K382 of p53 could suppress p53 binding to SLC7A11 promoter (Fig. 6M). These data suggested that FXR competitively binds with CBP to attenuate the inhibition of p53 on SLC7A11 expression, thereby inhibiting ferroptosis.

### FXR antagonist Z-GS induces ferroptosis and exerts antitumor efficacy via regulating p53/SLC7A11 pathway in vivo

**Fig. 7:**
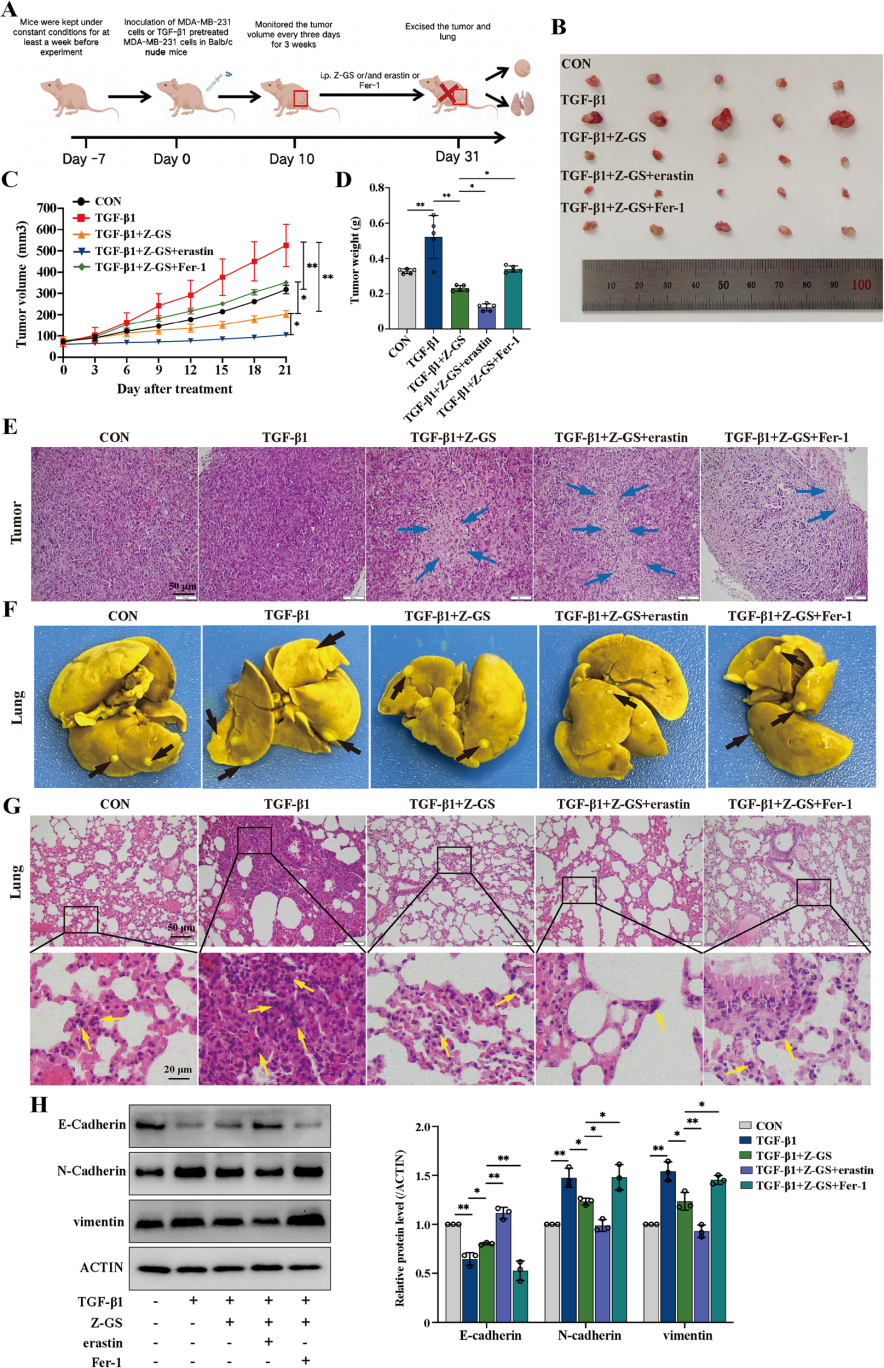
FXR antagonist Z-GS induces ferroptosis and exerts antitumor efficacy via regulating p53/SLC7A11 pathway in vivo. **A** An outline of the tumor inoculation and systemic injection process was shown. **B** An image of a xenograft tumor. **C**, **D** Tumor volume and tumor weight in each group. **E** Tumor necrosis was determined by H&E staining assay. The blue arrows indicate necrosis in the tumor. **F** The black arrows indicate the metastatic nodules in the lung tissue preserved in Bouin’s solution. **G** Tumor metastasis was determined by H&E staining assay. The yellow arrows indicate tumor metastasis in the lung tissue. **H** The expression levels of E-Cadherin, N-Cadherin, and vimentin in mixed xenograft tumors were analyzed by western blotting; n = 5 samples. Cont., control. The data are expressed as the mean ± SD (n = 5); ^*^p ≤ 0.05, ^**^p ≤ 0.01, and ^***^p ≤ 0.001.

To assess the therapeutic potential of Z-GS in vivo, we established experimental procedures (Fig. 7A). According to the in vivo results, combined treatment of TGF-β1 plus Z-GS significantly reduced tumor volume and weight compared to the TGF-β1-treated group. This effect was enhanced by the ferroptosis inducer erastin and blocked by the ferroptosis inhibitor Fer-1 (Fig. 7B–D). H&E staining of tumors also indicated that Z-GS might cause more cell deaths in the TGF-β1-pretreated group (Fig. 7E). Moreover, in the TGF-β1-treated group, primary tumors at the injection site and lung node metastases were significantly observed. The treatment of Z-GS effectively alleviated TGF-β1-induced pulmonary metastasis, with erastin enhancing the effect of Z-GS, while Fer-1 displayed the opposite effect (Fig. 7F). These results were confirmed via H&E staining of pulmonary tissues (Fig. 7G). Furthermore, western blotting analysis also revealed that Z-GS upregulated the expression level of E-cadherin and downregulated the expression levels of N-cadherin and vimentin (Fig. 7H).

To assess the potential side effects of Z-GS in vivo, we analyzed the liver and kidney indices, as well as ALT and Cr levels in serum. Various organs were harvested for H&E staining. There were no histological differences in the liver and kidney of the Z-GS-treated mice, suggesting that Z-GS has no significant toxicity in vivo (Fig. S6).

**Fig. 8:**
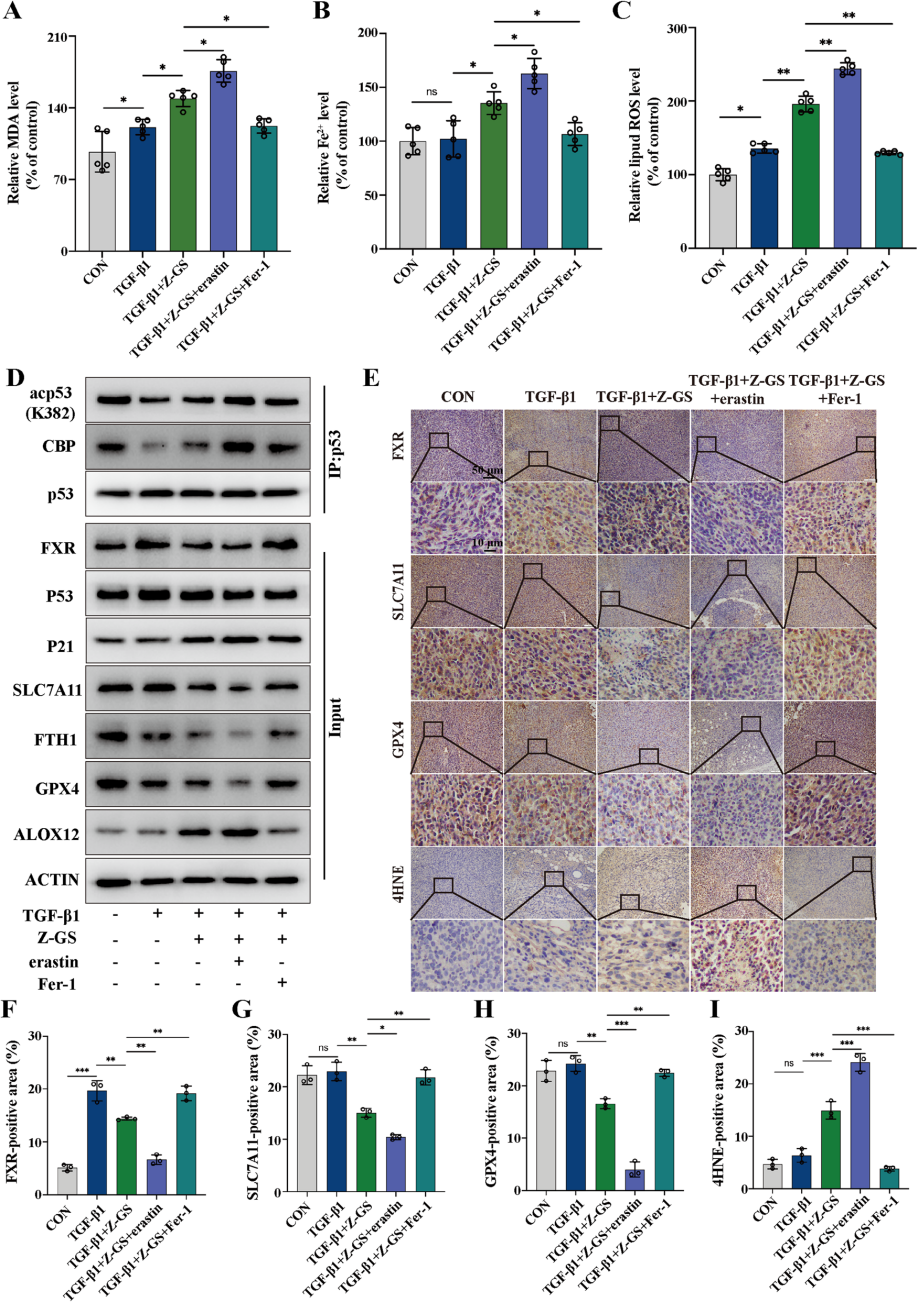
The mechanism of Z-GS-induced ferroptosis in xenograft mouse models. **A**–**C** Commercial kits were used to assess the relative levels of MDA, Fe^2+^, and lipid ROS in tumor tissues. **D** Tumor tissue lysates were collected and subjected to immunoprecipitation followed by immunoblotting analysis. Representative bands are shown. **E** IHC staining was performed to examine the expression levels of FXR, SLC7A11, GPX4 and 4HNE in tumor tissues and (**F**–**I**) the quantifications of IHC staining were shown; n = 5 samples. Cont., control. The data are expressed as the mean ± SD (n = 5); *p ≤ 0.05 and ^**^p ≤ 0.01.

We also explored the mechanism of Z-GS-induced ferroptosis in the mouse xenograft models. Firstly, we assessed several indicators of ferroptosis in tumor tissues, including MDA, Fe^2+^ and ROS levels. The results showed that the levels of MDA, Fe^2+^ and lipid ROS were increased after treatment with the combination of Z-GS and TGF-β1 compared to the TGF-β1-treated group. This effect was reversed by the administration of Fer-1 (Fig. 8A–C), suggesting that ferroptosis was involved in Z-GS-induced antitumor efficacy. As expected, immunohistochemical staining analysis showed that Z-GS decreased the expression levels of FXR, SLC7A11 and GPX4, and promoted the expression of 4HNE (Fig. 8E–I). Moreover, we detected the expression levels of p53, acetyl-p53(K382), and p21 by CoIP. Z-GS treatment promoted the protein expression of p21 as well as acetyl-p53 (K382), but not p53 itself, while this effect was significantly reversed by the administration of Fer-1. Meanwhile, the results revealed that Z-GS treatment increased the interaction between p53 and CBP and promoted p53 acetylation, indicating that the CBP-dependent p53 acetylation might be responsible for Z-GS-induced ferroptosis in the mouse xenograft models (Fig. 8D). In summary, our results demonstrated that Z-GS-induced ferroptosis inhibited TGF-β1-induced breast tumor growth and metastasis through the regulation of the CBP-dependent p53 acetylation pathway in vivo.

## Discussion

Ferroptosis is a new type of non-apoptotic regulatory cell death, caused by unrestricted lipid peroxidation and rupture of the plasma membrane [29]. However, the detailed mechanism of the regulation of ferroptosis in breast cancer is unclear. FXR, a nuclear receptor involved in lipid and glucose metabolism [30], has been shown to exhibit differential expression in cancer cells, closely correlating with cancer development and progression. For instance, high FXR expression has been associated with EMT progress and metastasis in hepatocellular carcinoma [31]. In this study, we unveiled the link between FXR and ferroptosis during breast carcinogenesis. Our findings suggested that FXR competitively binds with CBP, thereby reducing p53 acetylation at lysine 382. This, in turn, upregulated SLC7A11, a critical factor in ferroptosis. Consequently, the inhibition of FXR, either through Z-GS treatment or siRNA, was associated with anticancer effects via the induction of ferroptosis through the modulation of the CBP-dependent p53 acetylation. Our results filled the knowledge gap about that FXR acted as a novel biomarker for the diagnosis, prognosis, and therapy of breast cancer.

Increasing evidence revealed that FXR acts as a tumor promoter by activating carcinogenesis processes such as EMT, metastasis, and drug resistance [9], aligning with our observations in TGF-β1-induced cell and mouse metastatic models. The statistical analysis of 101 clinical samples of patients with breast carcinoma also revealed that FXR was associated with metastasis and poor prognosis. Moreover, we found that FXR deficiency suppressed TGF-β1-induced EMT and migration in breast cancer cells, as well as inhibited tumor growth and metastasis in nude mice. However, the mechanism by which FXR regulates the metastasis of breast cancer is not fully understood. Recent evidence demonstrated that FXR activation reduced PPARγ expression in the liver and induced antioxidant activity, leading to reducing liver lipid accumulation and steatosis [32]. Additionally, GW4064, an FXR agonist, can prevent liver iron accumulation and alleviate metabolic syndrome induced by iron overload [33]. As expected, treatment with Z-GS or FXR-siRNA led to increased levels of lipid ROS, ROS, MDA, and Fe^2+^ in breast cancer cells. This was accompanied by decreased expression of negative regulatory proteins of ferroptosis SLC7A11, FTH1, and GPX4, and increased the expression levels of ferroptosis-related protein ALOX12 and 4HNE. These results were further confirmed through the use of the ferroptosis inducer erastin and the ferroptosis inhibitor Fer-1. Our findings suggested that FXR deficiency was associated with the induction of ferroptosis in breast cancer, both in vitro and in vivo.

p53 is a well-established tumor suppressor gene that primarily functions as a transcription factor to activate or inhibit downstream target genes, leading to cell cycle arrest, DNA repair, cell senescence, apoptosis, and ferroptosis [34, 35]. Previous reports have suggested that acetylation of lysine at residue 373 (or position 382) of the p53 C-terminus enhances p53 activity and stability, enhancing the sensitivity of cells to oxidative stress responses [36]. In line with this, p53-siRNA transfection in our study indicated that the p53/SLC7A11 signaling pathway may be activated in breast cancer cells after siFXR treatment. Notably, the CoIP assays revealed no direct binding between FXR and p53, and the subcellular localization of p53 remained unaffected by FXR. Subsequent investigations strongly suggested that FXR deficiency triggers ferroptosis primarily through the activation of p53 acetylation at Lys382, leading to a significant decrease in the expression of SLC7A11 in vitro and in vivo. CBP is known to regulate the stability and transactivation of p53 in response to cellular stress, functioning as a tumor suppressor in cancer progression [37, 38]. Emerging evidences showed that the acetylation of lysine residues in the C-terminal region of p53 by CBP can promote the activation of p53 transcription [38–40]. More importantly, our data demonstrated that FXR inhibited nuclear p53 K382 acetylation by binding with CBP to suppress the interaction between p53 and CBP in the nucleus, accompanied with the restoration of the SLC7A11 expression. Therefore, FXR plays a crucial role in negatively regulating ferroptosis through modulation of the CBP-dependent p53 acetylation, ultimately inhibiting breast cancer metastasis.

In summary, our study demonstrated that the FXR deficiency significantly inhibited the proliferation and metastasis of breast cancer by inducing ferroptosis. The competitive binding of FXR with CBP was a crucial event that reduces p53 acetylation at lys382, alleviating its inhibitory effect on SLC7A11 and ultimately protecting cancer cells from ferroptosis. The FXR-regulated ferroptosis via modulation of the CBP-dependent p53 acetylation represents a novel mechanism and provides a potential therapeutic target for metastatic breast cancer.

## Materials and methods

### Antibodies and reagents

Z-Guggulsterone (HY-110066), Erastin (HY-15763), and Ferrostatin-1 (HY-100579) were procured from MedChemExpress (NJ, USA). Z-VAD-FMK (GC12861), Necrosulfonamide (NSA, GC10150), and 3-Methyladenine (3-MA, GC10710) were procured from Glpbio Co. (CA, USA). Recombinant human transforming growth factor-β1 (TGF-β1) was supplied by Peprotech (Rocky Hill, NJ, USA). Polyclonal antibodies against FXR (25055-1-AP), E-cadherin (20874-1-AP), N-cadherin (22018-1-AP), vimentin (10366-1-AP), p53 (10442-1-AP), p53 (60283-2-Ig), GPX4 (30388-1-AP), SLC7A11 (26864-1-AP), β-actin (20536-1-AP), EIF4E (29712-1-AP), HRP-conjugated Affinipure Goat Anti-Rabbit IgG (SA00001-2) and HRP-conjugated Affinipure Goat Anti-Mouse IgG (SA00001-1-A) were obtained from Proteintech (Wuhan, China). Polyclonal antibody against FTH1 (A19544) was purchased from Abclonal (Wuhan, China). Polyclonal antibodies against ALOX12 (ab168384), acetyl-p53 (acetyl K305)(ab109396), acetyl-p53 (acetyl K370)(ab183544), acetyl-p53 (acetyl K373)(ab62376) and acetyl-p53 (acetyl K382)(ab75754) were obtained from Abcam (UK). Monoclonal antibody against Flag was purchased from Cell Signaling Technology (14793, MA, USA). Monoclonal antibodies against CBP(sc-365387) and FXR(sc-25309) were purchased from Santa Cruz Biotechnology (CA, USA).

### Clinical specimens

Breast cancer tumors and matched adjacent normal tissues were collected from patients at the First Affiliated Hospital of Nanchang University between April 1, 2013, and January 1, 2022. Supplementary Table 1 summarized the clinical information of 101 patients with breast carcinoma in this study. Patients were excluded if they met the following criteria: non-breast cancer patients, missing clinical and pathological data, or death due to other causes after an operation. This study was approved by the Ethics Committee of the First Affiliated Hospital of Nanchang University (NO. (2023)CDYFYYLK(08021)).

### Immunohistochemistry (IHC) staining

The staining procedure was performed as previously described [41]. Primary antibodies used in this study included FXR, SLC7A11, vimentin, GPX4 and 4HNE. Staining intensity was scored as follows: 0 = no staining, 1 = moderate staining, 2 = intense staining, 3 = very intense staining. The percentage of immunostaining-positive cells were categorized into four groups: 0 = 0%, 1 = 1–25%, 2 = 26–50%, 3 = 51–75%, 4 = 76–100%. The immunohistochemical score was calculated by multiplying the staining intensity score by the percentage of immunostaining-positive cells.

### Cell culture

MCF-7(RRID:CVCL_0031) cells and MCF-10A(RRID:CVCL_0598) cells were obtained from Procell Life Science & Technology Co., Ltd(Wuhan, China). MDA-MB-231(RRID:CVCL_0062) cells were purchased from Shanghai Anwei Biotechnology Co., Ltd. Cells were cultured in Dulbecco’s modified Eagle medium (DMEM, Solarbio, Beijing, China) supplemented with 10% fetal bovine serum (FBS, Biological Industries, Israel) at 37 °C with 5% CO_2_.

### siRNA and plasmid transfection

The human small interfering RNAs targeting CBP, FXR and p53 were formulated and synthesized by KeyGEN BioTECH (Jiangsu, China). The sequences of siRNAs were shown in Supplementary Table 3. Plasmids of Flag-FXR, Myc-CBP and control vector were purchased from KeyGEN BioTECH (Jiangsu, China). HA-p53 and HA-p53-K382R plasmid were purchased from Hunan Fenghui Biotechnology Co., Ltd (Hunan, China). The protein and nucleic acid sequences of TP53-WT and TP53-K382R were shown in Supplementary Table 4. The siRNA or plasmid and Lipofectamine^TM^ 3000 were diluted to a specific concentration in Opti-MEM medium, mixed, and incubated following the manufacturer’s instructions.

### Cell viability assay

Cell viability was assessed using the Cell Counting Kit-8 (CCK8, GlpBio, USA). Cells were plated in 96-well plates at a density of 5000 cells per well. Cell viability was measured using a CCK8 Assay Kit after drug treatment and determined based on the absorbance at 490 nm, then measured using a microplate reader.

### Cell colony forming assay

To determine cell colony-forming ability, MCF-7 and MDA-MB-231 cells were treated with Z-GS at different concentrations and seeded into 6-well plates at a density of 1000 cells/mL. The drug-containing medium was changed every two days, and after two weeks, colonies were fixed, stained, photographed and counted.

### Transwell and wound-healing assay

For the cell migration assay, 4 × 10^4^ cells were seeded into upper chambers with serum-free medium, while the lower chambers contained medium with 10% FBS as a chemoattractant, then divided into different groups: CON, TGF-β1 group (10 ng/mL TGF-β1 pretreated for 24 h), TGF-β1 + 20 μM Z-GS group (10 ng/mL TGF-β1 pretreated for 24 h, then treated with 20 μM Z-GS for 24 h), and TGF-β1 + 40 μM Z-GS group (10 ng/mL TGF-β1 pretreated for 24 h, then treated with 40 μM Z-GS for 24 h). For the cell invasion assay, matrix glue was diluted with serum-free medium, and 100 µL was added to the upper chamber and incubated at 37 °C for 1 h. The subsequent steps were similar to the transwell migration assay. Cells that migrated and invaded the lower surface were fixed, stained, and photographed with a light microscope (Olympus, Japan).

For the wound-healing assay, a confluent monolayer of cells at 95% confluence was mechanically scratched using a 200 µL pipette tip and subsequently cultured in serum-free medium to observe wound closure.

### Western blotting

Proteins were extracted using RIPA lysis containing protease inhibitors (P0100, Solarbio, Beijing, China). Protein separation was performed via 8-10% SDS-PAGE, followed by electroblotting onto polyvinylidene fluoride membranes. Primary antibodies used were E-cadherin (1:2 000), N-cadherin (1:4 000), vimentin (1:3 000), p53 (1:5 000), GPX4 (1:2 000), SLC7A11 (1:2 000), ALOX12 (1:1 000), FTH1 (1:2 000), acetyl-p53 (K305, 1:1 000), acetyl-p53 (K370, 1:1 000), acetyl-p53 (K373, 1:1 000), acetyl-p53 (K382, 1:1 000), CBP (1:1 000), and β-actin (1:5 000). Immunoreactive bands were analyzed using a chemiluminescence western blotting detection system, and quantification was performed using ImageJ software.

### Quantitative real-time PCR

Total RNA was extracted from the cells using commercial kits (Aidlab, Beijing) according to the manufacturer’s instructions. The SYBR Green PCR Master Mix (AQ601, TransGen Biotech, Beijing, China) was used for quantitative real-time PCR (qRT-PCR) and relative mRNA expressions were standardized to GAPDH. The primers for FXR and GAPDH were synthesized by Generay Biotech (Shanghai, China). The sequences were shown in Supplementary Table 2. QPCR cycling conditions included predenaturing at 94 °C for 30 s, followed by 45 cycles (94 °C for 5 s, 60 °C for 15 s and 72 °C for 10 s) and extension at 72 °C for 30 s. The relative expression levels were calculated by 2^-∆∆Ct^ method.

### Co-immunoprecipitation (CoIP)

The cells were treated with IP lysis buffer (Thermo, USA) containing protease inhibitors for 15 min. They were then centrifuged at 12,000 × *g* for 10 min at 4 °C. The supernatants were incubated overnight at 4 °C with specific antibodies, followed by an additional hour at 25 °C with protein A/G beads (Thermo Fisher Scientific, Catalog No. 88802). Finally, the supernatants were boiled in SDS-loading buffer and subjected to western blotting analysis.

### BOPIDY staining and confocal image

We assessed the levels of lipid ROS in breast cancer cells using C11-BODIPY ^581/591^ (GC40165, Lipid Peroxidation, GLPBio, USA) according to the manufacturer’s instructions. After treating the cells with different agents for 24 h, they were washed twice with PBS and incubated with C11-BODIPY^581/591^ (5 µM) for 30 min at 37 °C. Subsequently, the nuclei were stained with DAPI for 5 min. Analysis was conducted using a multifunctional enzyme labeling instrument (PerkinElmer, USA) and a confocal laser scanning microscopy system (Nikon, Japan). The ratio of oxidation type to reduction type represented the oxidation intensity, where reduced products exhibited red fluorescence at 530/590 nm and oxidized products exhibited green fluorescence at 485/528 nm.

### Cellular ROS, ferrous ion and lipid peroxidation

Intracellular ROS levels were detected using the fluorescent probe DCFH-DA (S0033, Beyotime Biotechnology, Shanghai, China). The level of Fe^2+^ was measured using an iron colorimetric assay kit (E-BC-K773, Elabscience, Wuhan, China). Lipid peroxidation, assessed by measuring MDA, a major indicator, was determined using a commercial kit (S0131S, Beyotime Biotechnology, Shanghai, China). All procedures were carried out as previously described [42] and followed the manufacturer’s instructions.

### Transmission electron microscopy (TEM)

Cell specimens were fixed in 0.1 M phosphate buffer (PB) containing 2.5% glutaraldehyde overnight at 4 °C. The samples were then dehydrated with increasing ethanol and acetone concentrations, embedded in EPON812, and polymerized for 48 h at 60 °C. Ultrathin sections were sliced using a Leica Ultracut microslicer (Leica, USA), followed by staining with uranyl acetate and led citrate for 15 min at room temperature. Images were captured using a transmission electron microscope (HITACHI, Japan).

### Confocal microscopy experiment

Sterilized round slides are placed on a 12-well plate. Breast cancer cells were digested and resuspended in the seeding plate and were then fixed with 4% paraformaldehyde (PFA) for 20 min. To visualize cytoplasmic and nuclear components, cells were permeabilized with 0.25% Triton X-100 for 20 min, followed by blocking with 5% BSA for 1 h and incubation with relevant antibodies overnight at 4 °C. After PBS washing, the cells were incubated with fluorescently labeled secondary antibodies for 1 h at room temperature. Finally, an anti-fluorescence quenching sealing tablet was used to seal the cells, and images were acquired using a confocal laser scanning microscopy system (Nikon, Tokyo, Japan).

### Dual-luciferase reporter assay

Lipofectamine^TM^ 3000 reagent (Invitrogen, Carlsbad, CA, USA) was used to transfect cells in a 24-well plate with the SLC7A11 promoter and other target plasmids (HA-p53, HA-p53 K382R, Flag-FXR, sip53, siCBP, siFXR). To detect dual luciferase activity, cells were prepared according to the Dual-Luciferase Reporter Assay Kit (RG029, Beyotime Biotechnology, Shanghai, China) protocols.

### Chromatin immunoprecipitation (ChIP)

ChIP was conducted using a ChIP assay kit (P2078, Beyotime Biotechnology, Shanghai, China) according to the manufacturer’s instructions. The primer sequences targeting the SLC7A11 promoter region were as follows:

Forward: 5ʹ-TTGAGCAACAAGCTCCTCCT-3ʹ;

Reverse: 5ʹ-CAAACCAGCTCAGCTTCCTC-3ʹ.

### Tumor xenografts in nude mice

Six-week-old female Balb/c nude mice were obtained from Hunan SJA Laboratory Animals Co., Ltd (Hunan, China) under Permission No. SCXK 2019-0004 and were housed in SPF facilities. A suspension of 1 × 10^7^/mL MDA-MB-231 cells in 200 µL of serum-free DMEM medium (mixed with Matrigel) were injected on the second mammary fat pad of nude mice. After the xenografts reached 100 mm^3^, mice were randomly divided into groups as follows: (1) control group; (2) TGF-β1 group (MDA-MB-231 cells pre-treated with 10 ng/mL TGF-β1 for 24 h before injection); (3) TGF-β1 + Z-GS group (MDA-MB-231 cells pre-treated with 10 ng/mL TGF-β1 for 24 h before injection, and 30 mg/kg Z-GS intraperitoneal injection every three days); (4) TGF-β1 + Z-GS+erastin group (MDA-MB-231 cells pre-treated with 10 ng/mL TGF-β1 for 24 h before injection, and 30 mg/kg Z-GS and 20 mg/kg erastin intraperitoneal injection every three days); and (5) TGF-β1 + Z-GS+Fer-1 group (MDA-MB-231 cells pre-treated with 10 ng/mL TGF-β1 for 24 h before injection, and 30 mg/kg Z-GS and 1 mg/kg Fer-1 intraperitoneal injection every three days). Tumor volume was calculated using the formula: (Length × Width^2^)/2. Further analysis of tumors and lungs was conducted after sacrificing the mice 21 days after injection. The animal experiments were approved by Nanchang University’s Animal Ethics Committee (NO. NCUSYDWFL-202126, 7th March, 2021).

### Hematoxylin and eosin (H&E) staining

We fixed, dehydrated, and embedded the tumor, lung, kidney, and liver tissues in paraffin. We sliced the sections with a microtome to a thickness of (4 µm), then placed them in a water bath at 42 °C, unfolded the sections, removed them, and baked them at 60 °C for later use. The tissues were subsequently deparaffinized, stained with a hematoxylin-eosin solution, followed by dehydration and sealing. Finally, we viewed and photographed the stained sections using a microscope (Olympus, Japan).

### Statistical analysis

Statistical analyses were performed using GraphPad Prism 9 (GraphPad Software, San Diego, CA, USA) and SPSS 22.0 software (SPSS Inc., Chicago, IL, USA) through Student’s *t* test, one-way ANOVA or two-way ANOVA. A *p* value less than 0.05 was considered statistically significant (*p ≤ 0.05, **p ≤ 0.01, ***p ≤ 0.001 and ****p ≤ 0.0001). All values are expressed as the mean ± standard error.

## Supplementary information

Supplementary materials

checklist

Original Western Blots

## Data Availability

Data that form the basis of this report are available upon request.
