# Supplementary material for: FXR deficiency induced ferroptosis via modulation of the CBP-dependent p53 acetylation to suppress breast cancer growth and metastasis
Source: Cell Death Dis. 2024 Nov 14;15(11):826. doi: 10.1038/s41419-024-07222-3 (PMC11564727; doi:10.1038/s41419-024-07222-3)
Supplement: Supplementary file 1 — Supplementary materials [file 41419_2024_7222_MOESM1_ESM.docx]

**Supplementary Information for**

**FXR deficiency induced ferroptosis via** **modulation of the** **CBP-dependent** **p53 acetylation to suppress breast cancer growth and metastasis**

**Ping Huang^1#^, Han Zhao^1#^, Hua Dai^3#^,** **Jinying Li^1^, Xiafang Pan^1^, Wentian Pan^1^, Chunhua Xia^1,2*^**^*^ **and Fanglan Liu^1,2*^**

^1^School of Pharmacy, Jiangxi Medical College, Nanchang University, Nanchang 330006, P. R. China.

^2^Jiangxi Province Key Laboratory of New Drug Evaluation and Transformation, Nanchang 330031, P. R. China.

^3^Department of Pathology, the First Affiliated Hospital of Nanchang University, Nanchang 330038, P. R. China.

^*^Correspondence: [liufanglan@ncu.edu.cn](mailto:liufanglan@ncu.edu.cn) for Fanglan Liu. School of Pharmacy, Jiangxi Medical College, Nanchang University Xuefu road 1299, Nanchang, P. R. China

^**^Co-correspondence: xch720917[@ncu.edu.cn](mailto:liufanglan@ncu.edu.cn) for Chunhua Xia. School of Pharmacy, Jiangxi Medical College, Nanchang University Xuefu road 1299, Nanchang, P. R. China

^#^Ping Huang, Han Zhao and Hua Dai contributed equally to this work.

**Supplementary Figures**

**Figure S1**


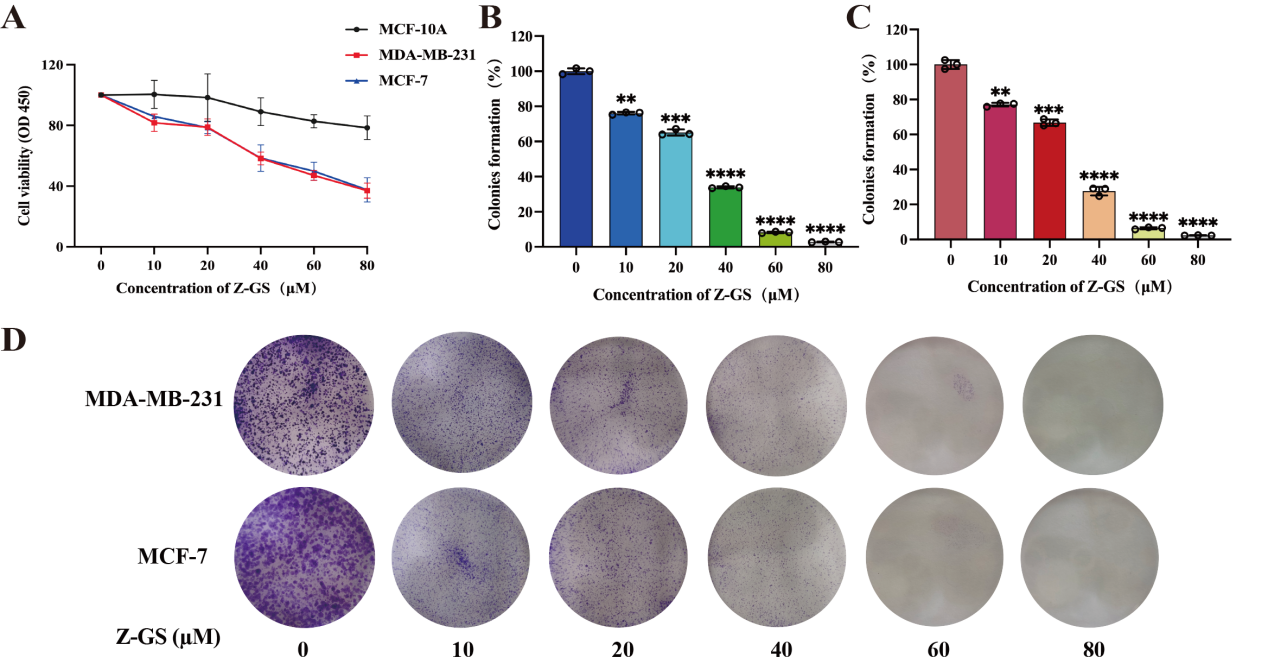


**Figure S1. FXR antagonist Z-GS inhibits cell proliferation and migration of** **breast cancer cells.** (A) MCF-10A, MDA-MB-231 and MCF-7 cells were treated with different concentrations of Z-GS for 24 h, and cell viability was evaluated by the CCK-8 assay; n = 5 samples. (B-D) Colony formation and quantitative analysis of MDA-MB-231 and MCF-7 cells treated with different concentrations of Z-GS. Z-GS, 0, 10, 20, 40, 60 and 80 µM; n = 3 samples.The data are expressed as the mean ±SD; *^**^p* ≤ 0.01, *^***^p* ≤ 0.001 and *^****^p* ≤ 0.0001.

**Figure S2**


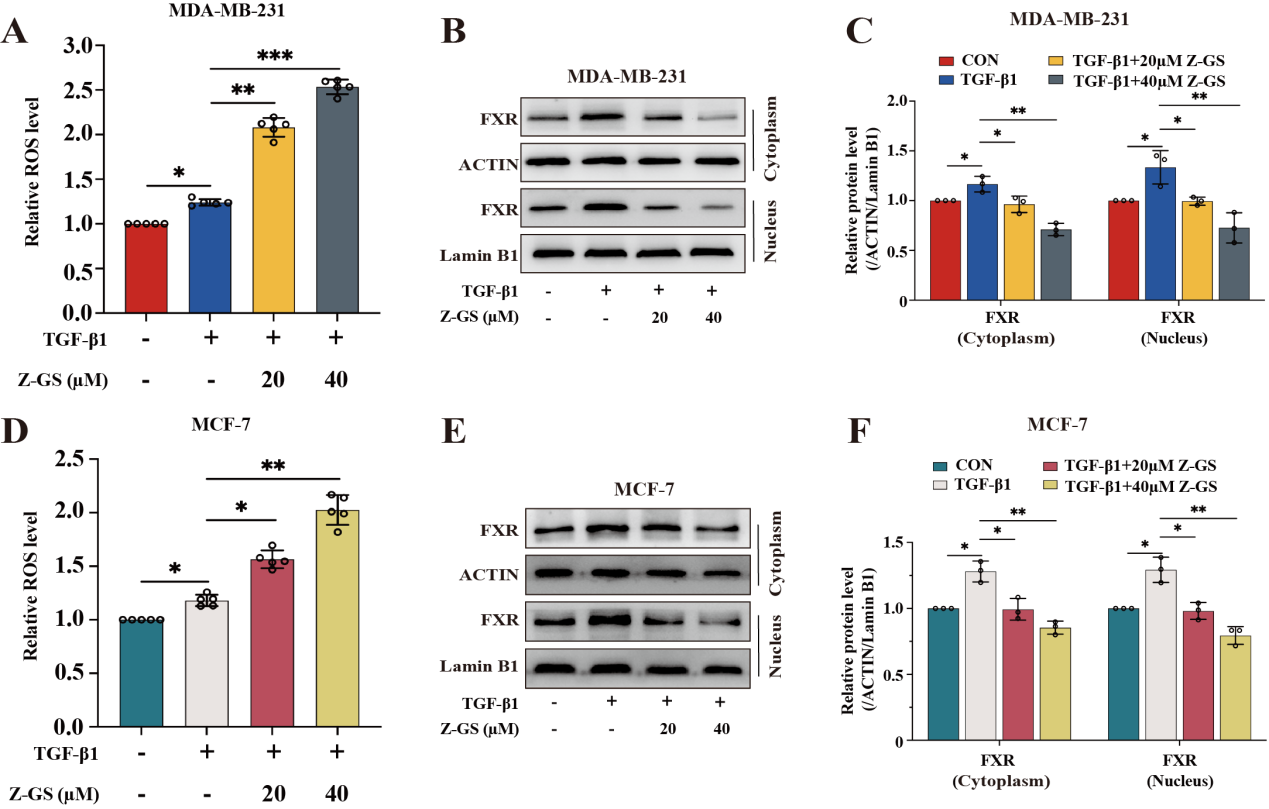


**Figure S2. Z-GS inhibits the expression of FXR and** **leads to elevated levels of ROS** in breast cancer cells. (A&D) Cellular ROS production was evaluated using a 2′,7′-dichlorofluorescein diacetate (DCFDA) kit; n = 5 samples. (B&E) The expression of FXR in the cytoplasm and nucleus was examined by western blotting assays in MDA-MB-231 cells and MCF-7 cells; (C&F) The quantification of protein expression of FXR in MDA-MB-231 cells and MCF-7 cells were calculated; n = 3 samples. Cont., control; TGF-β1, 10 ng/mL; Z-GS, 20 and 40 µM. The data are expressed as the mean ±SD; *^*^p* ≤ 0.05, *^**^p* ≤ 0.01, *^***^p* ≤ 0.001 and *^****^p* ≤ 0.0001.

**Figure S3**


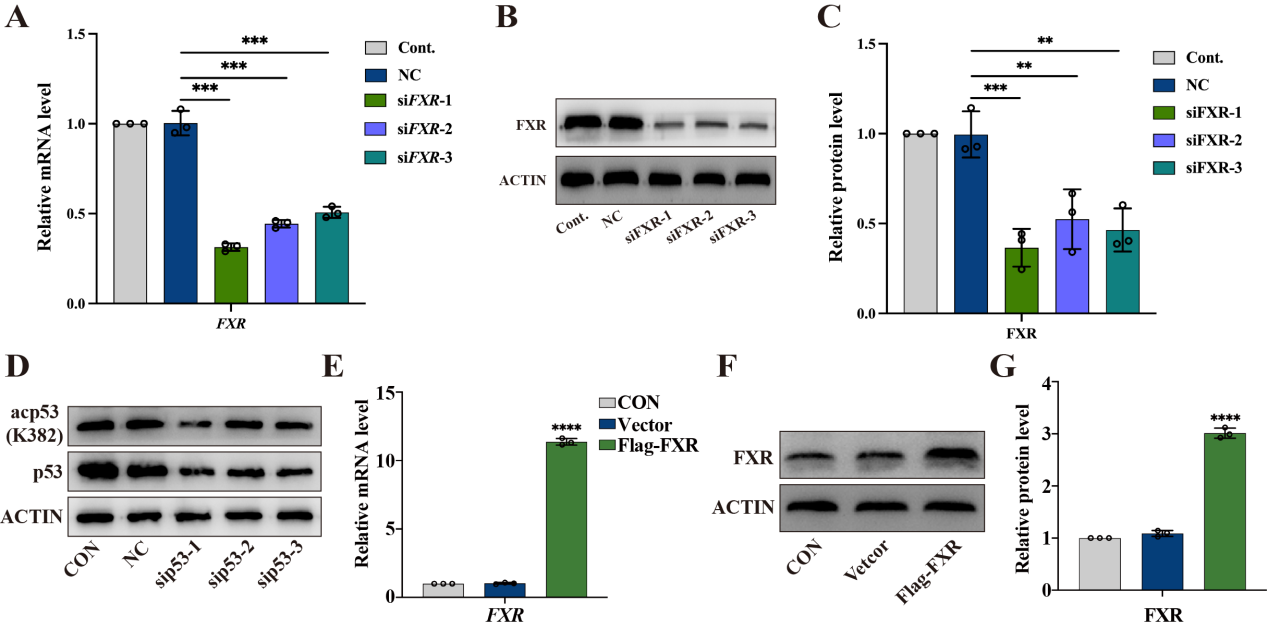


### **Figure S3.** **The [effects of RNA interference-mediated knockdown of](https://webofscience.clarivate.cn/wos/woscc/full-record/WOS:000322207500033) targeting genes expression.** (A-C) Western blotting assays and qPCR assays were used to detect the efficiency of transfection of siFXR. (D) Western blotting assays was used to detect the efficiency of transfection of sip53. (E-G) Western blotting assays and qPCR assays were used to detect the efficiency of transfection of FXR. n = 3 samples. Cont., control. The data are expressed as the mean ±SD; ***p* ≤ 0.01, ****p* ≤ 0.001 and *****p* ≤ 0.0001.

**Figure S4**


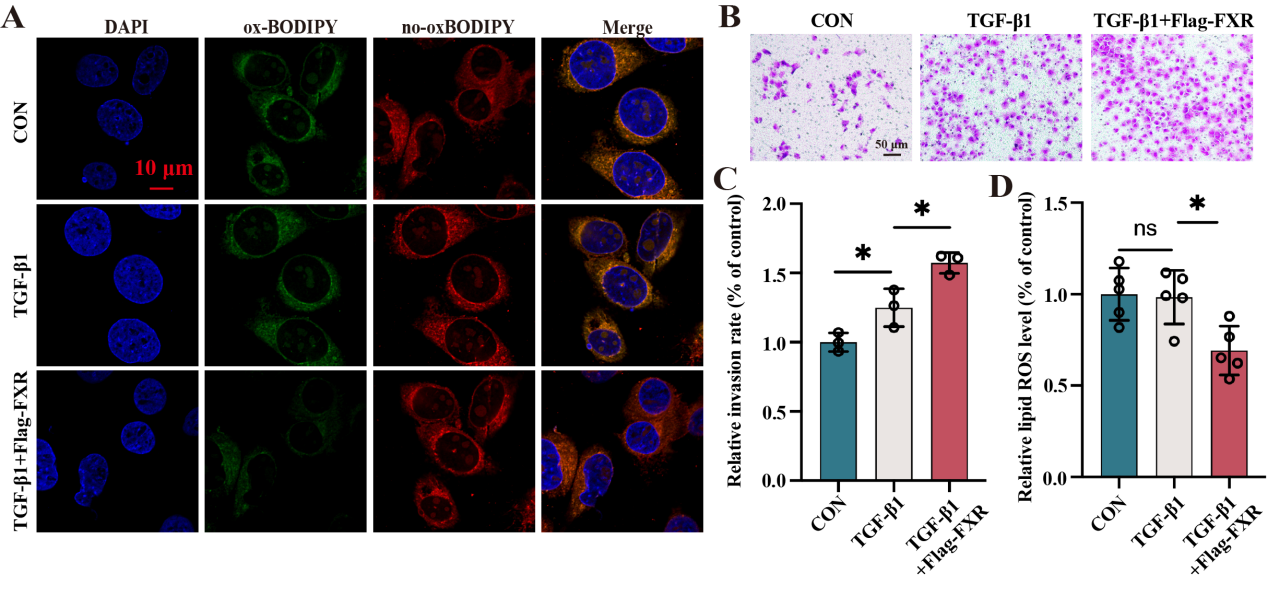


**Figure S4. FXR overexpression promotes TGF-β1-induced invasion and migration of breast cancer cells.** (A&D) The relative levels of lipid ROS were detected by the C11-BODIPY probe, and the images were captured using a fluorescence microscope. (B) Transwell migration assay was performed to assess the migration ability of MDA-MB-231 cells. (C) The quantification of transwell migration assay. n = 3 samples. Cont., control; TGF-β1, 10 ng/mL. The data are expressed as the mean ±SD; *^*^p* ≤ 0.05.

**Figure S5**

**
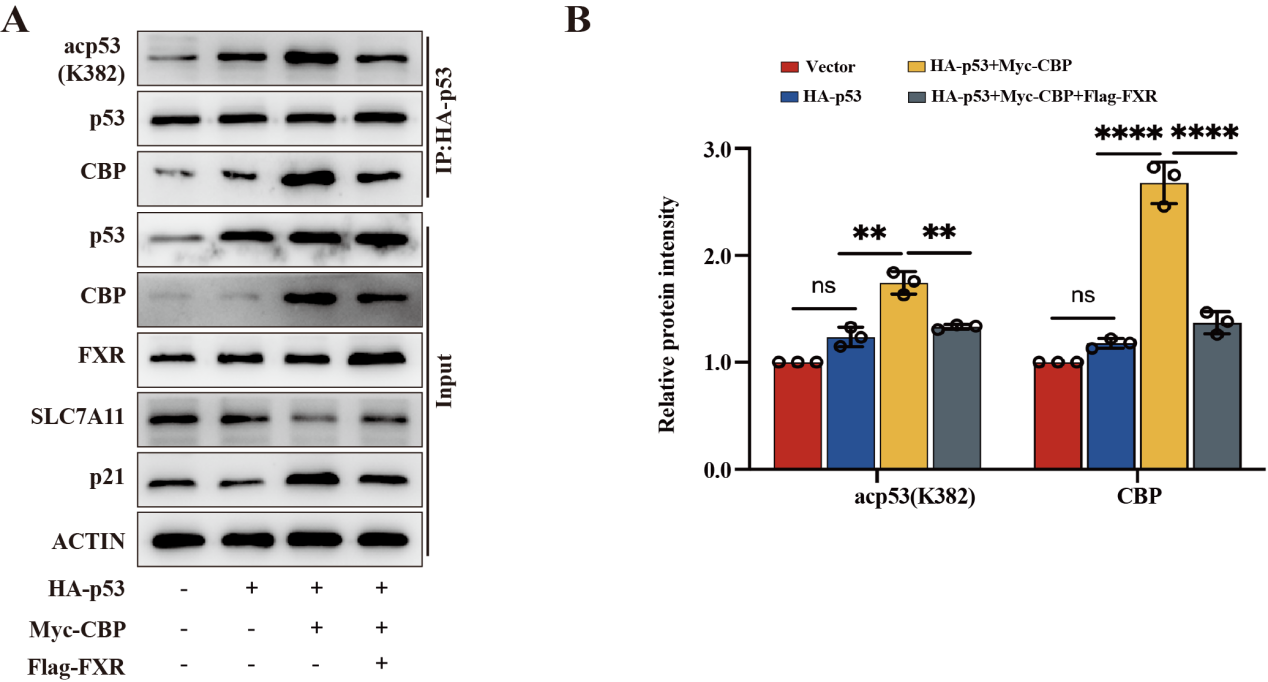
**

**Figure S5. FXR and p53 compete for CBP binding.** (A&B) MDA-MB-231 cells were transfected with HA-p53, Myc-CBP and Flag-FXR and harvested for CoIP with an anti-p53 antibody; n = 3 samples; The data are expressed as the mean ±SD; *^**^p* ≤ 0.01 and *^****^p* ≤ 0.0001.

**Figure S6**


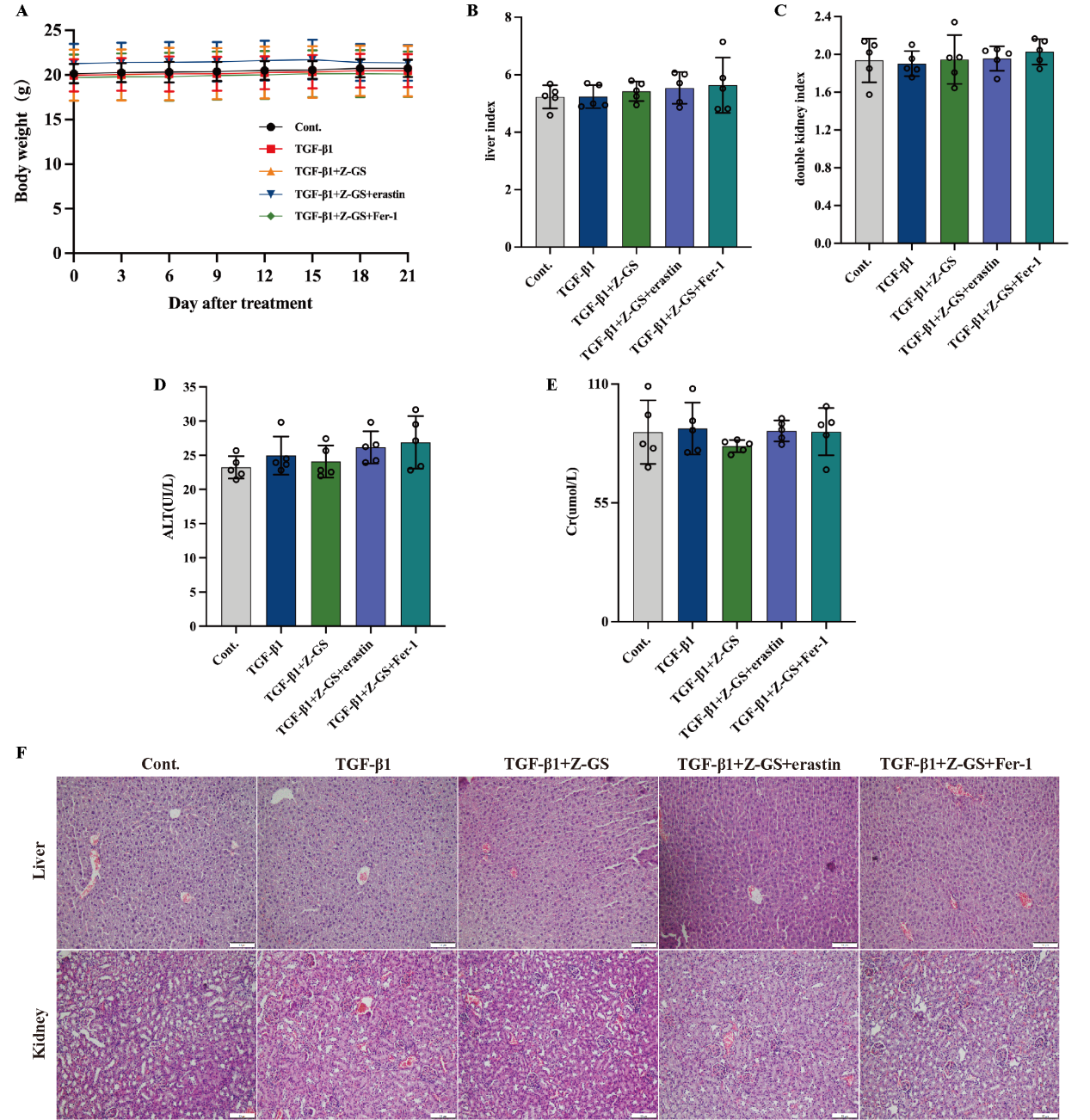


**Figure S6. The effects of Z-GS on liver and kidney toxicity in nude mice.** (A) The body weight of nude mice was measured every three days. (B&C) The liver index and double kidney index in nude mice. (D&E) The relative levels of ALT and Cr in the serum were assessed using commercial kits. (F) H&E staining sections of the liver and kidney were analyzed; n = 5 samples. Cont., control. The data are expressed as the mean ±SD (n=5).

**Supplementary Table 1. Clinicopathological patient characteristics of 101 Breast Cancer Patients**

| Characteristics | Total | FXR expression | | *P* value | *χ*^2^ value | Vimentin  expression | | *P* value | *χ*^2^  value | SLC7A11  expression | | *P* value | *χ*^2^  value |
| --- | --- | --- | --- | --- | --- | --- | --- | --- | --- | --- | --- | --- | --- |
|  |  | High | Low |  |  | High | Low |  |  | High | Low |  |  |
| Age |  |  |  | 0.682 | 0.168 |  |  | 0.659 | 0.195 |  |  | 0.932 | 0.007 |
| ≤60 years | 81 | 57 | 24 |  |  | 61 | 20 |  |  | 60 | 21 |  |  |
| >60 years | 20 | 15 | 5 |  |  | 16 | 4 |  |  | 15 | 5 |  |  |
| Tumor differentiation |  |  |  | 0.720 | 0.658 |  |  | 0.391 | 1.879 |  |  | 0.644 | 0.881 |
| Poor | 35 | 24 | 11 |  |  | 24 | 11 |  |  | 25 | 10 |  |  |
| moderate | 58 | 43 | 15 |  |  | 47 | 11 |  |  | 43 | 15 |  |  |
| Well | 8 | 5 | 3 |  |  | 6 | 2 |  |  | 7 | 1 |  |  |
| Tumor invasion |  |  |  | 0.911 | 0.013 |  |  | 0.772 | 0.084 |  |  | 0.393 | 0.728 |
| T1-2 | 90 | 64 | 26 |  |  | 69 | 21 |  |  | 68 | 22 |  |  |
| T3-4 | 11 | 8 | 3 |  |  | 8 | 3 |  |  | 7 | 4 |  |  |
| Lymph node metastasis |  |  |  | 0.003 | 8.997 |  |  | 0.017 | 5.663 |  |  | 0.001 | 21.551 |
| N0 | 46 | 26 | 20 |  |  | 30 | 16 |  |  | 24 | 22 |  |  |
| N1-N3 | 55 | 46 | 9 |  |  | 47 | 8 |  |  | 51 | 4 |  |  |
| Distant metastasis |  |  |  | 0.033 | 4.534 |  |  | 0.033 | 4.557 |  |  | 0.004 | 8.216 |
| M0 | 76 | 50 | 26 |  |  | 54 | 22 |  |  | 51 | 25 |  |  |
| M1 | 25 | 22 | 3 |  |  | 23 | 2 |  |  | 24 | 1 |  |  |
| TNM stage（AJCC） |  |  |  | 0.033 | 4.534 |  |  | 0.033 | 4.557 |  |  | 0.019 | 5.471 |
| Ⅰ-Ⅱ | 76 | 50 | 26 |  |  | 54 | 22 |  |  | 52 | 24 |  |  |
| Ⅲ-Ⅳ | 25 | 22 | 3 |  |  | 23 | 2 |  |  | 23 | 2 |  |  |

**Supplementary Table 2. Sequences for qPCR primers**

| **Gene** | **Base sequence (5’-3’)** |
| --- | --- |
| Human GAPDH-forward | 5′-AGGAGAGGACTTCGACAACCG-3′ |
| Human GAPDH-reverse | 5′-CAGGTCCTTCCCATGCTTCC-3′ |
| Human FXR-forward | 5′-TGTGTCCATGAGGCACAGAG-3′ |
| Human FXR-reverse | 5′-GTCGGGGAGACAATGAGGTG-3′ |

**Supplementary Table 3. The sequences of siRNAs**

| **Gene** | **Base sequence (5’-3’)** |
| --- | --- |
| Human CBP siRNA#1 | 5’-GGCCUCCUCAAUAGUAACUTT-3’ |
| Human CBP siRNA#2 | 5’-AGUUACUAUUGAGGAGGCCTT-3’ |
| Human CBP siRNA#3 | 5’-AACAGTGGGAACCTTGTTCCA-3’ |
| Human FXR siRNA#1 | 5’-AAUCUAAGCGACUGAGAAATT-3’ |
| Human FXR siRNA#2 | 5’-CAGAGAUGCCUGUAACAAATT-3’ |
| Human FXR siRNA#3 | 5’-CAAGUGACCUCGACAACAATT-3’ |
| Human p53 siRNA#1 | 5’-GCGCACAGAGGAAGAGAAUTT-3’ |
| Human p53 siRNA#2 | 5’-CCACUGGAUGGAGAAUAUUTT-3’ |
| Human p53 siRNA#3 | 5’-CCAUCCACUACAACUACAUTT-3’ |

**Supplementary Table 4. Protein and nucleic acid sequences of TP53-WT and TP53-K382R**

tumor protein p53 [ Homo sapiens (human) ]

Gene ID: 7157 NM_000546 1179bp

**TP53-WT：**

ATGGAGGAGCCGCAGTCAGATCCTAGCGTCGAGCCCCCTCTGAGTCAGGAAACATTTTCAGACCTATGGAAACTACTTCCTGAAAACAACGTTCTGTCCCCCTTGCCGTCCCAAGCAATGGATGATTTGATGCTGTCCCCGGACGATATTGAACAATGGTTCACTGAAGACCCAGGTCCAGATGAAGCTCCCAGAATGCCAGAGGCTGCTCCCCCCGTGGCCCCTGCACCAGCAGCTCCTACACCGGCGGCCCCTGCACCAGCCCCCTCCTGGCCCCTGTCATCTTCTGTCCCTTCCCAGAAAACCTACCAGGGCAGCTACGGTTTCCGTCTGGGCTTCTTGCATTCTGGGACAGCCAAGTCTGTGACTTGCACGTACTCCCCTGCCCTCAACAAGATGTTTTGCCAACTGGCCAAGACCTGCCCTGTGCAGCTGTGGGTTGATTCCACACCCCCGCCCGGCACCCGCGTCCGCGCCATGGCCATCTACAAGCAGTCACAGCACATGACGGAGGTTGTGAGGCGCTGCCCCCACCATGAGCGCTGCTCAGATAGCGATGGTCTGGCCCCTCCTCAGCATCTTATCCGAGTGGAAGGAAATTTGCGTGTGGAGTATTTGGATGACAGAAACACTTTTCGACATAGTGTGGTGGTGCCCTATGAGCCGCCTGAGGTTGGCTCTGACTGTACCACCATCCACTACAACTACATGTGTAACAGTTCCTGCATGGGCGGCATGAACCGGAGGCCCATCCTCACCATCATCACACTGGAAGACTCCAGTGGTAATCTACTGGGACGGAACAGCTTTGAGGTGCGTGTTTGTGCCTGTCCTGGGAGAGACCGGCGCACAGAGGAAGAGAATCTCCGCAAGAAAGGGGAGCCTCACCACGAGCTGCCCCCAGGGAGCACTAAGCGAGCACTGCCCAACAACACCAGCTCCTCTCCCCAGCCAAAGAAGAAACCACTGGATGGAGAATATTTCACCCTTCAGATCCGTGGGCGTGAGCGCTTCGAGATGTTCCGAGAGCTGAATGAGGCCTTGGAACTCAAGGATGCCCAGGCTGGGAAGGAGCCAGGGGGGAGCAGGGCTCACTCCAGCCACCTGAAGTCCAAAAAGGGTCAGTCTACCTCCCGCCATAAAAAACTCATGTTCAAGACAGAAGGGCCTGACTCAGAC

**TP53-WT: Amino acid sequence**

MEEPQSDPSVEPPLSQETFSDLWKLLPENNVLSPLPSQAMDDLMLSPDDIEQWFTEDPGPDEAPRMPEAAPPVAPAPAAPTPAAPAPAPSWPLSSSVPSQKTYQGSYGFRLGFLHSGTAKSVTCTYSPALNKMFCQLAKTCPVQLWVDSTPPPGTRVRAMAIYKQSQHMTEVVRRCPHHERCSDSDGLAPPQHLIRVEGNLRVEYLDDRNTFRHSVVVPYEPPEVGSDCTTIHYNYMCNSSCMGGMNRRPILTIITLEDSSGNLLGRNSFEVRVCACPGRDRRTEEENLRKKGEPHHELPPGSTKRALPNNTSSSPQPKKKPLDGEYFTLQIRGRERFEMFRELNEALELKDAQAGKEPGGSRAHSSHLKSKKGQSTSRHKKLMFKTEGPDSD

**TP53-K382R:**

ATGGAGGAGCCGCAGTCAGATCCTAGCGTCGAGCCCCCTCTGAGTCAGGAAACATTTTCAGACCTATGGAAACTACTTCCTGAAAACAACGTTCTGTCCCCCTTGCCGTCCCAAGCAATGGATGATTTGATGCTGTCCCCGGACGATATTGAACAATGGTTCACTGAAGACCCAGGTCCAGATGAAGCTCCCAGAATGCCAGAGGCTGCTCCCCCCGTGGCCCCTGCACCAGCAGCTCCTACACCGGCGGCCCCTGCACCAGCCCCCTCCTGGCCCCTGTCATCTTCTGTCCCTTCCCAGAAAACCTACCAGGGCAGCTACGGTTTCCGTCTGGGCTTCTTGCATTCTGGGACAGCCAAGTCTGTGACTTGCACGTACTCCCCTGCCCTCAACAAGATGTTTTGCCAACTGGCCAAGACCTGCCCTGTGCAGCTGTGGGTTGATTCCACACCCCCGCCCGGCACCCGCGTCCGCGCCATGGCCATCTACAAGCAGTCACAGCACATGACGGAGGTTGTGAGGCGCTGCCCCCACCATGAGCGCTGCTCAGATAGCGATGGTCTGGCCCCTCCTCAGCATCTTATCCGAGTGGAAGGAAATTTGCGTGTGGAGTATTTGGATGACAGAAACACTTTTCGACATAGTGTGGTGGTGCCCTATGAGCCGCCTGAGGTTGGCTCTGACTGTACCACCATCCACTACAACTACATGTGTAACAGTTCCTGCATGGGCGGCATGAACCGGAGGCCCATCCTCACCATCATCACACTGGAAGACTCCAGTGGTAATCTACTGGGACGGAACAGCTTTGAGGTGCGTGTTTGTGCCTGTCCTGGGAGAGACCGGCGCACAGAGGAAGAGAATCTCCGCAAGAAAGGGGAGCCTCACCACGAGCTGCCCCCAGGGAGCACTAAGCGAGCACTGCCCAACAACACCAGCTCCTCTCCCCAGCCAAAGAAGAAACCACTGGATGGAGAATATTTCACCCTTCAGATCCGTGGGCGTGAGCGCTTCGAGATGTTCCGAGAGCTGAATGAGGCCTTGGAACTCAAGGATGCCCAGGCTGGGAAGGAGCCAGGGGGGAGCAGGGCTCACTCCAGCCACCTGAAGTCCAAAAAGGGTCAGTCTACCTCCCGCCATAAAAGACTCATGTTCAAGACAGAAGGGCCTGACTCAGAC

**TP53-K382R: Amino acid sequence**

MEEPQSDPSVEPPLSQETFSDLWKLLPENNVLSPLPSQAMDDLMLSPDDIEQWFTEDPGPDEAPRMPEAAPPVAPAPAAPTPAAPAPAPSWPLSSSVPSQKTYQGSYGFRLGFLHSGTAKSVTCTYSPALNKMFCQLAKTCPVQLWVDSTPPPGTRVRAMAIYKQSQHMTEVVRRCPHHERCSDSDGLAPPQHLIRVEGNLRVEYLDDRNTFRHSVVVPYEPPEVGSDCTTIHYNYMCNSSCMGGMNRRPILTIITLEDSSGNLLGRNSFEVRVCACPGRDRRTEEENLRKKGEPHHELPPGSTKRALPNNTSSSPQPKKKPLDGEYFTLQIRGRERFEMFRELNEALELKDAQAGKEPGGSRAHSSHLKSKKGQSTSRHKRLMFKTEGPDSD
