# Supplementary material for: FXR deficiency induced ferroptosis via modulation of the CBP-dependent p53 acetylation to suppress breast cancer growth and metastasis
Source: Cell Death Dis. 2024 Nov 14;15(11):826. doi: 10.1038/s41419-024-07222-3 (PMC11564727; doi:10.1038/s41419-024-07222-3)

Full and uncropped western blot for Figure 3

Figure 3F

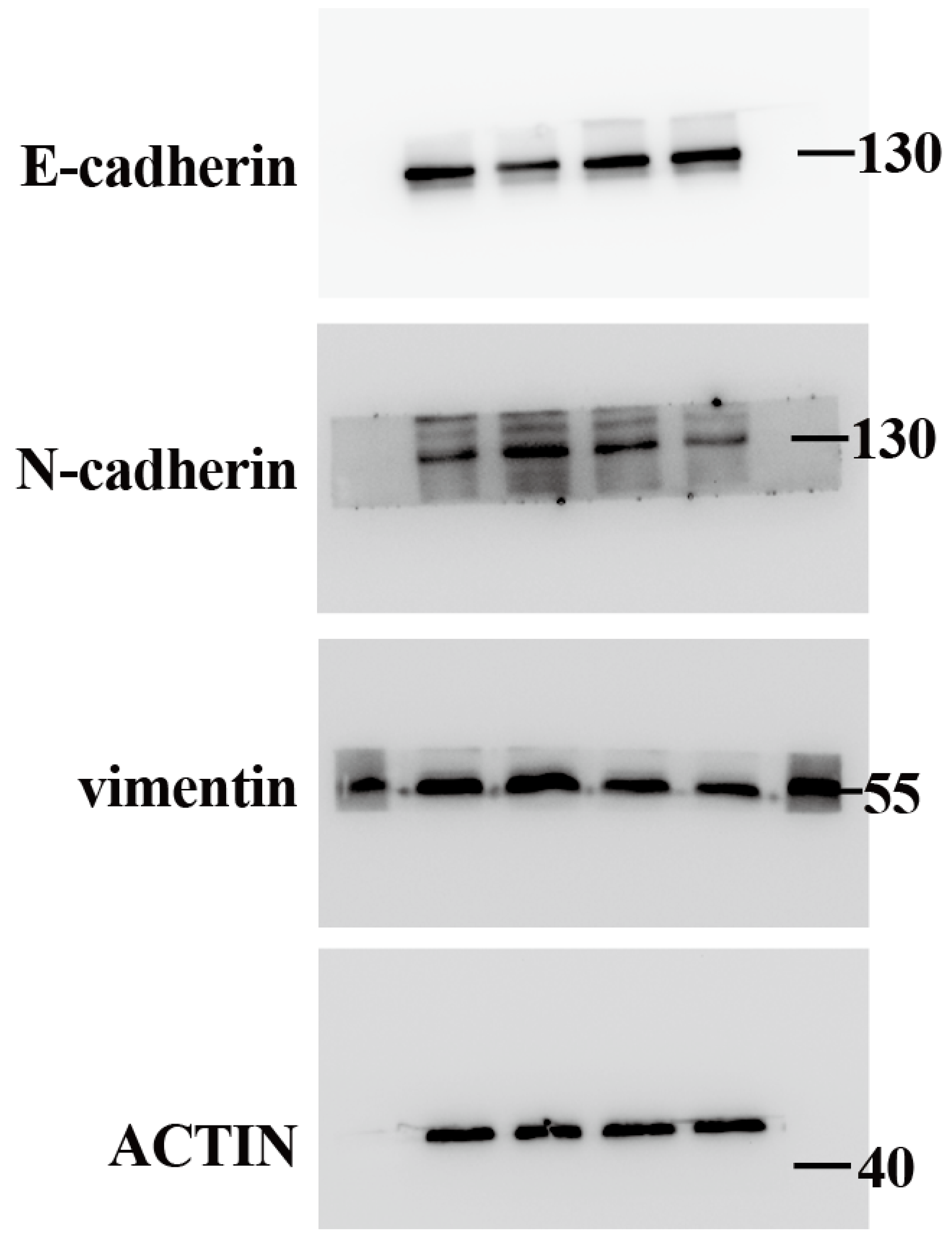

Figure 3G

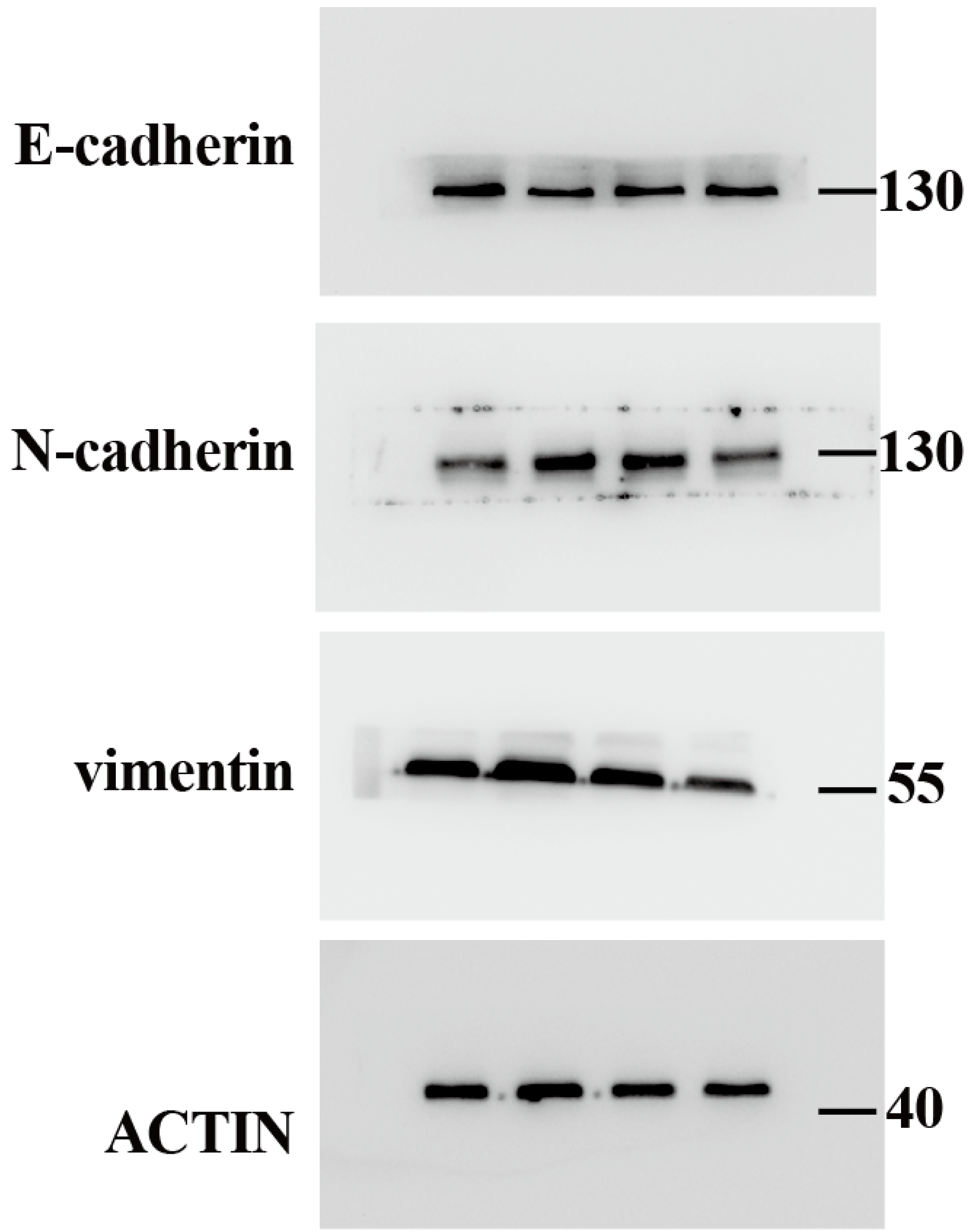

Figure 3R

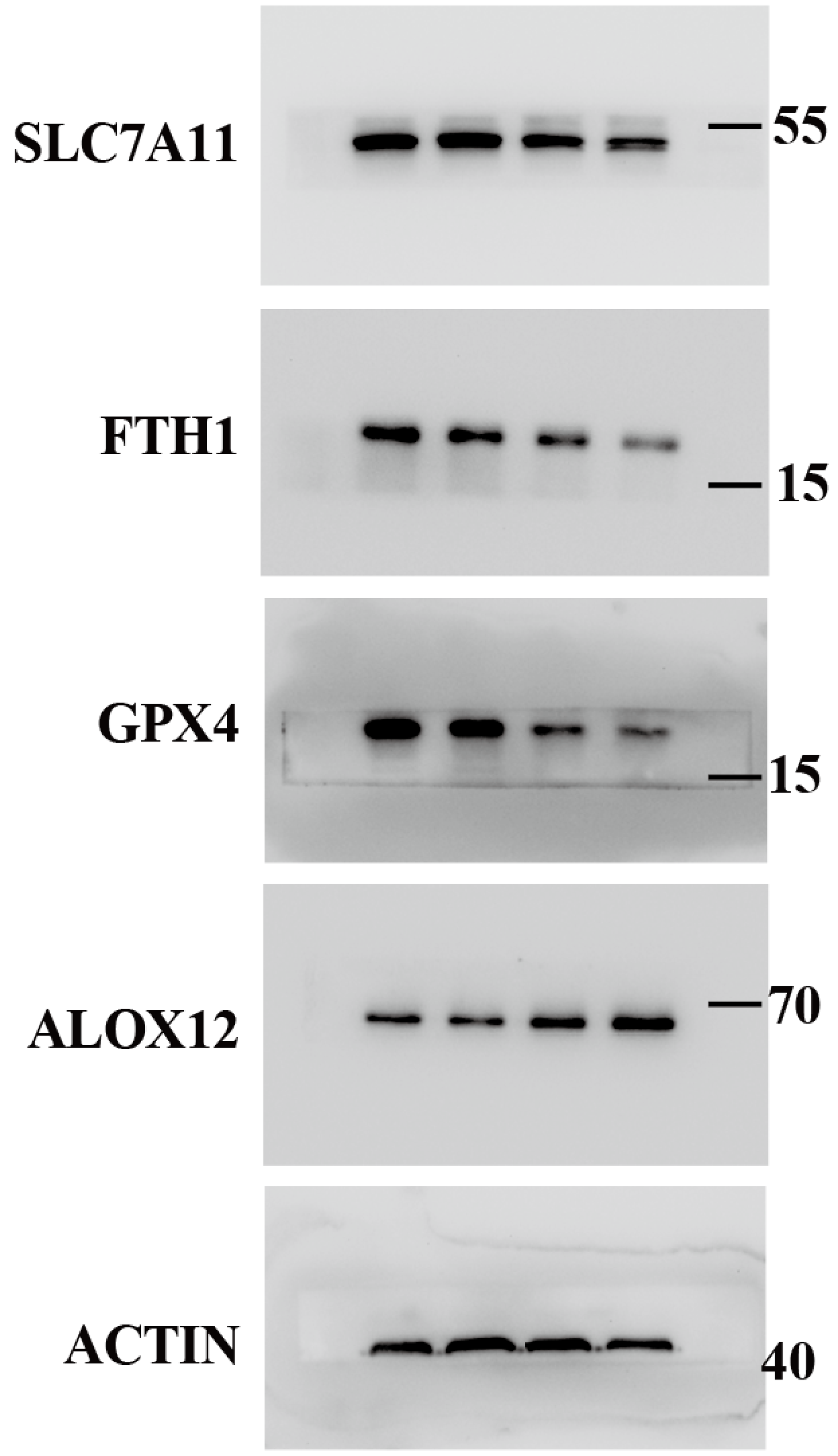

Figure 3S

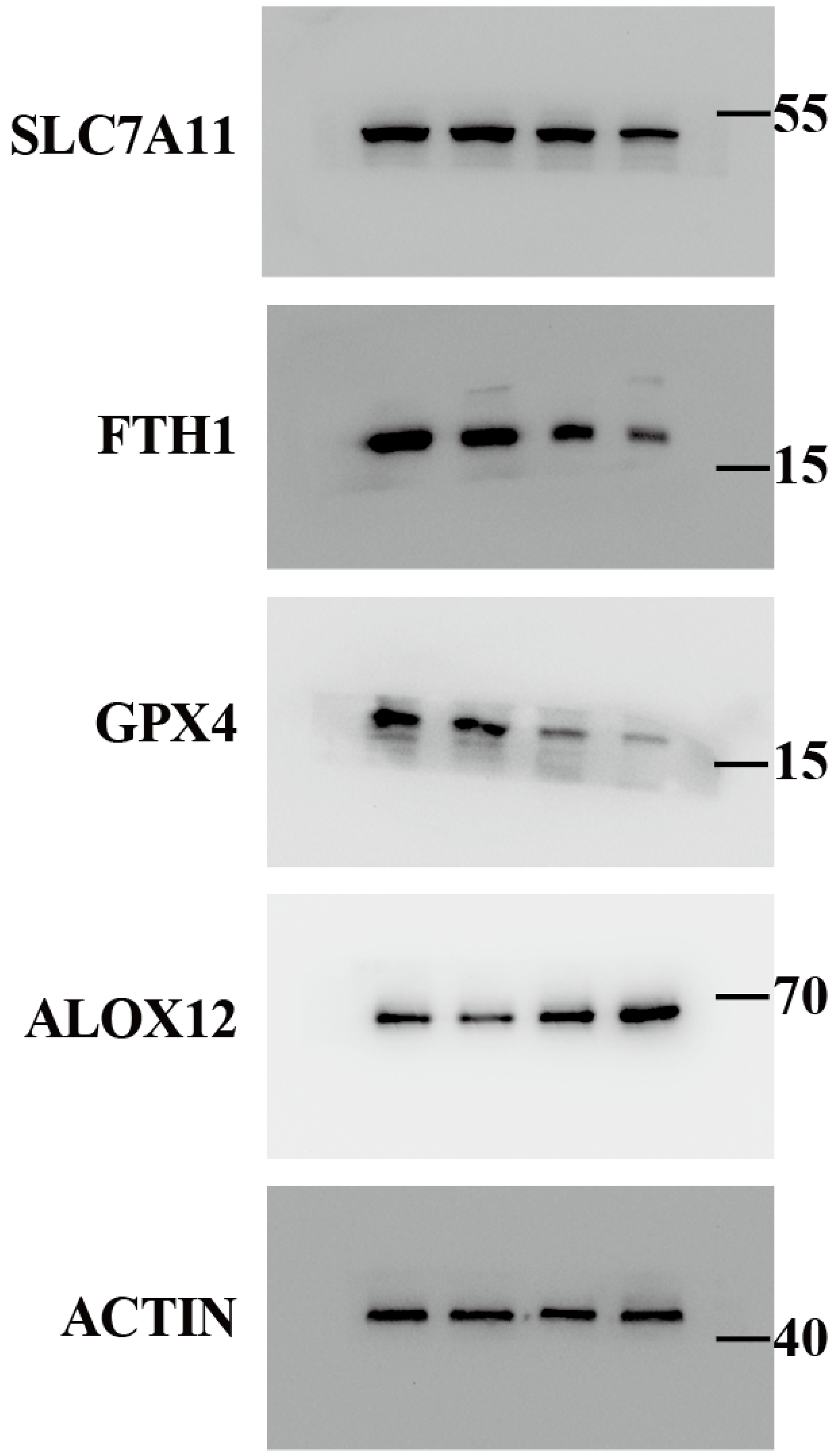

Full and uncropped western blot for Figure 4

Figure 4E

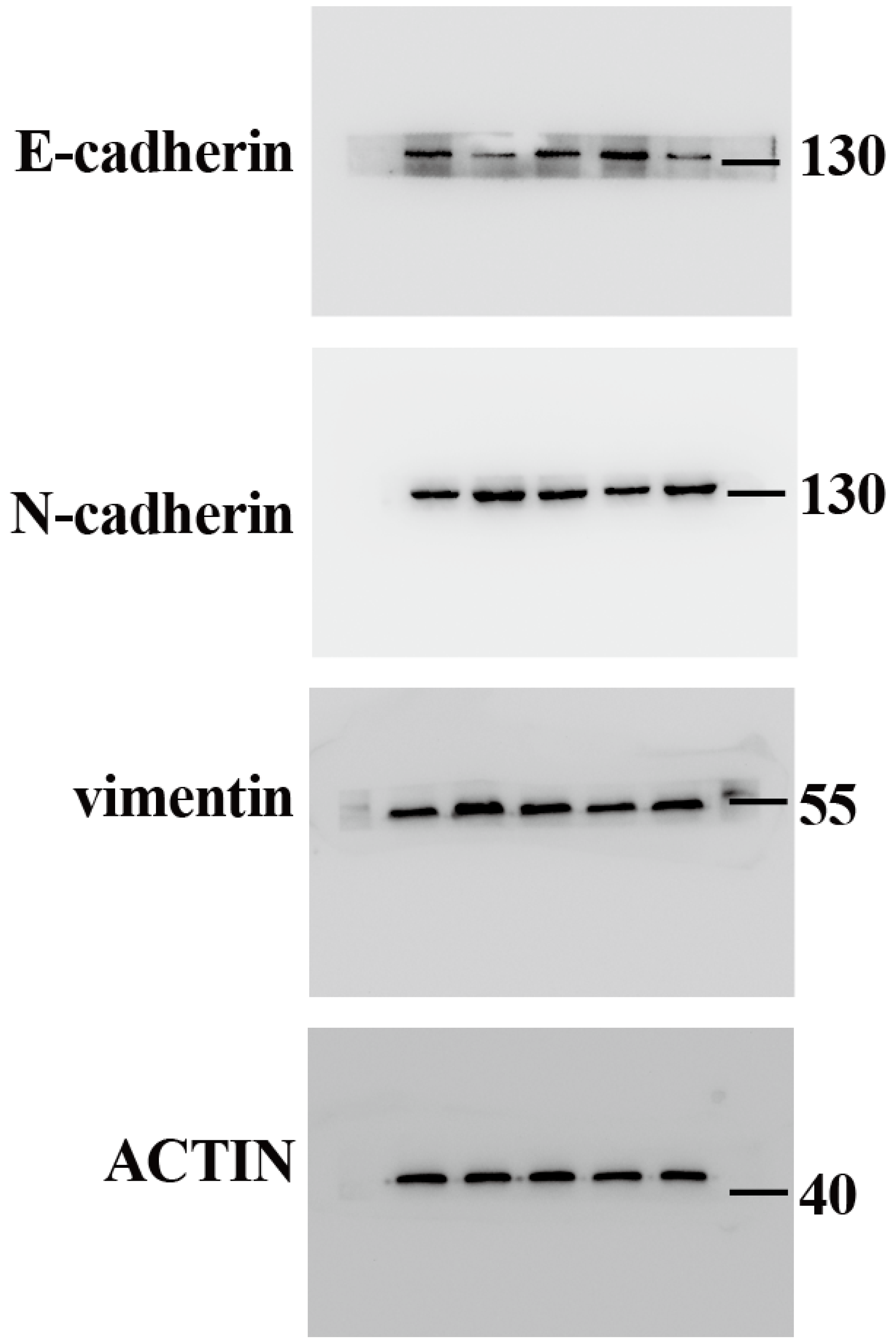

Figure 4J

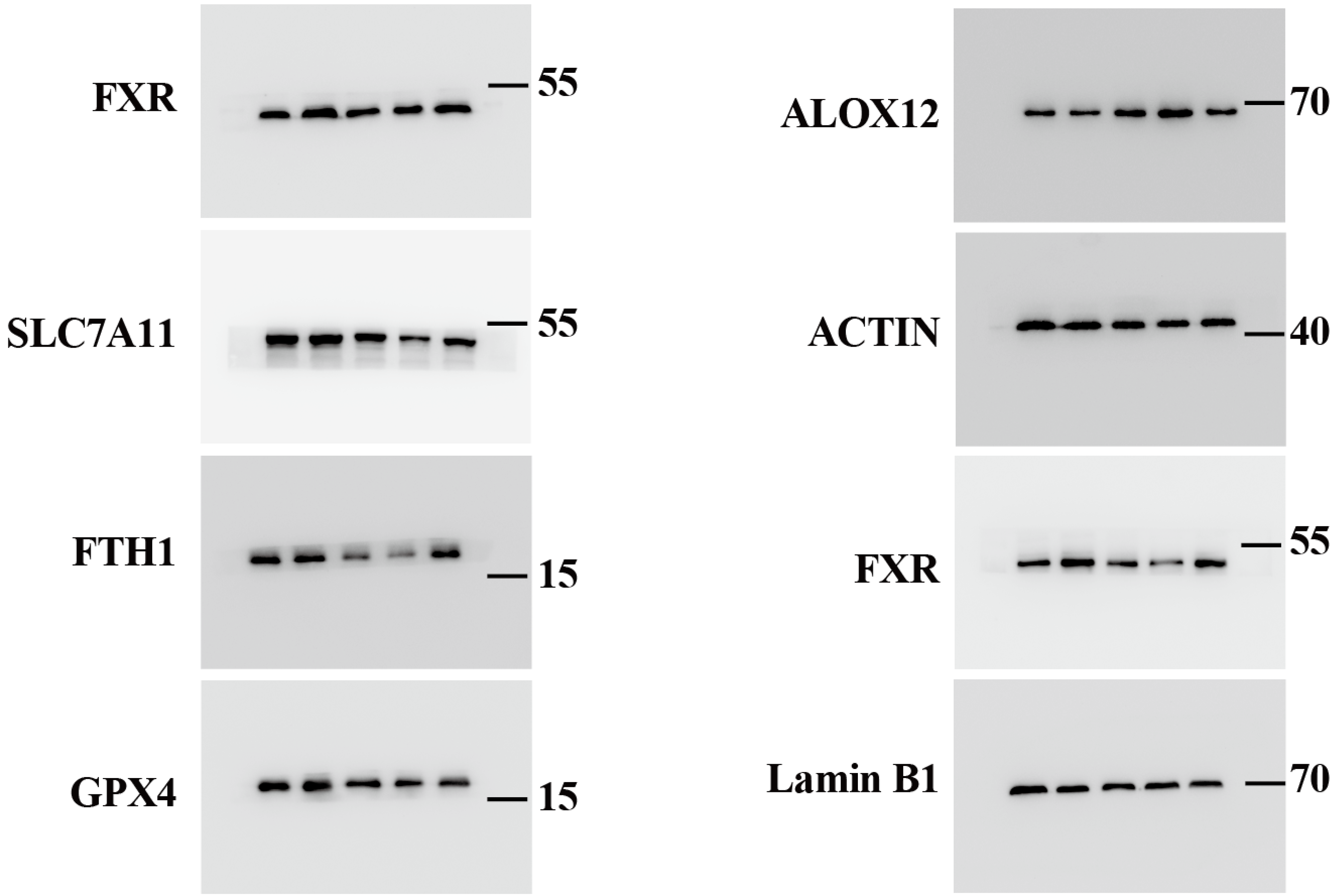

Full and uncropped western blot for Figure 5

Figure 5A

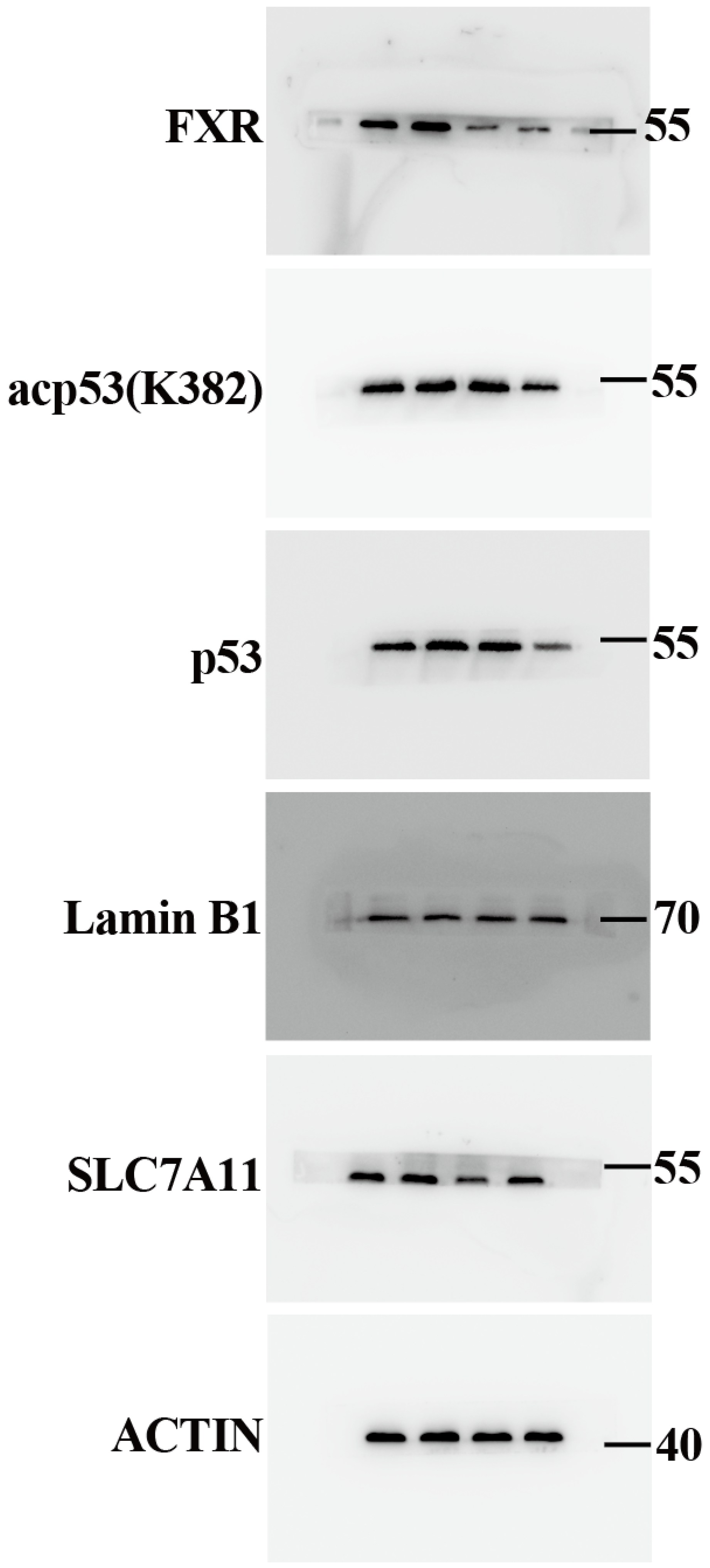

Figure 5G

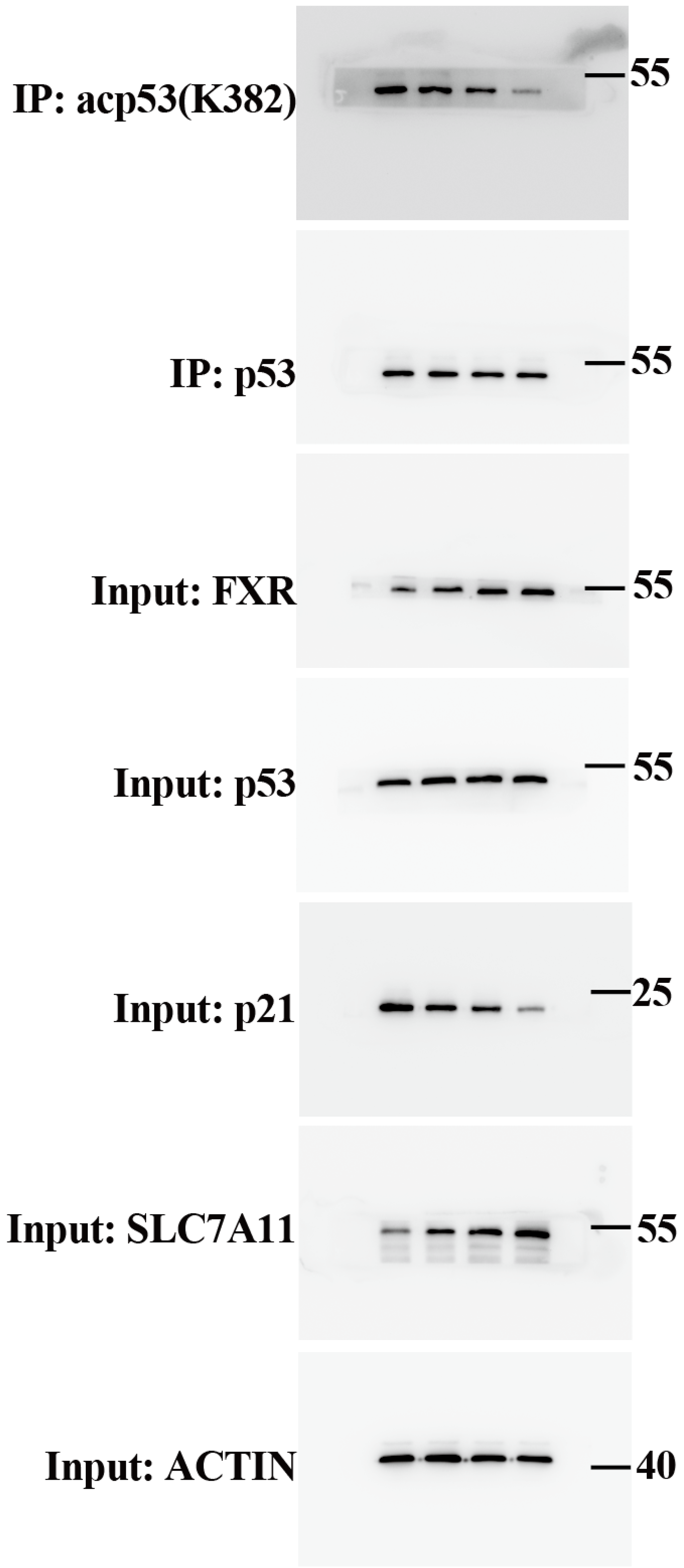

Figure 5I

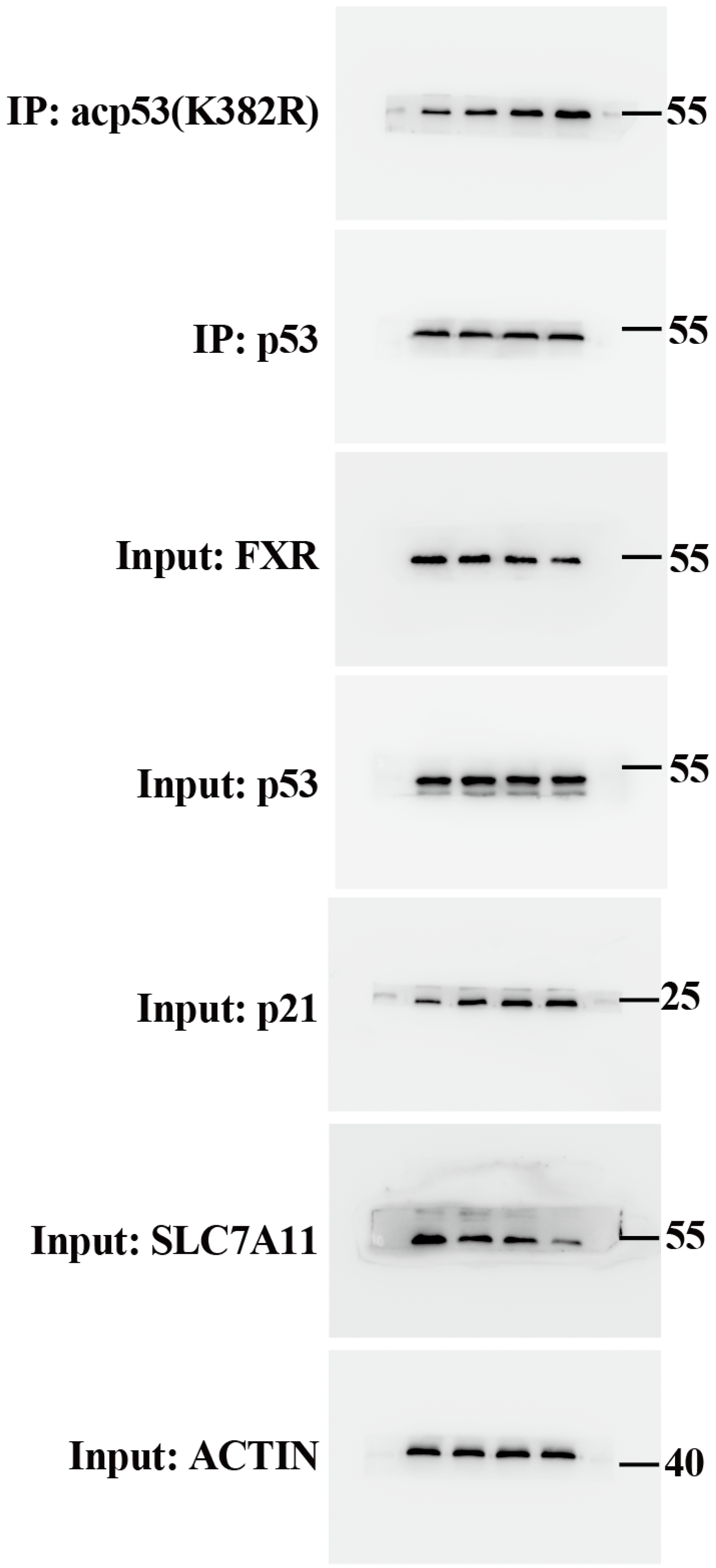

Figure 5K

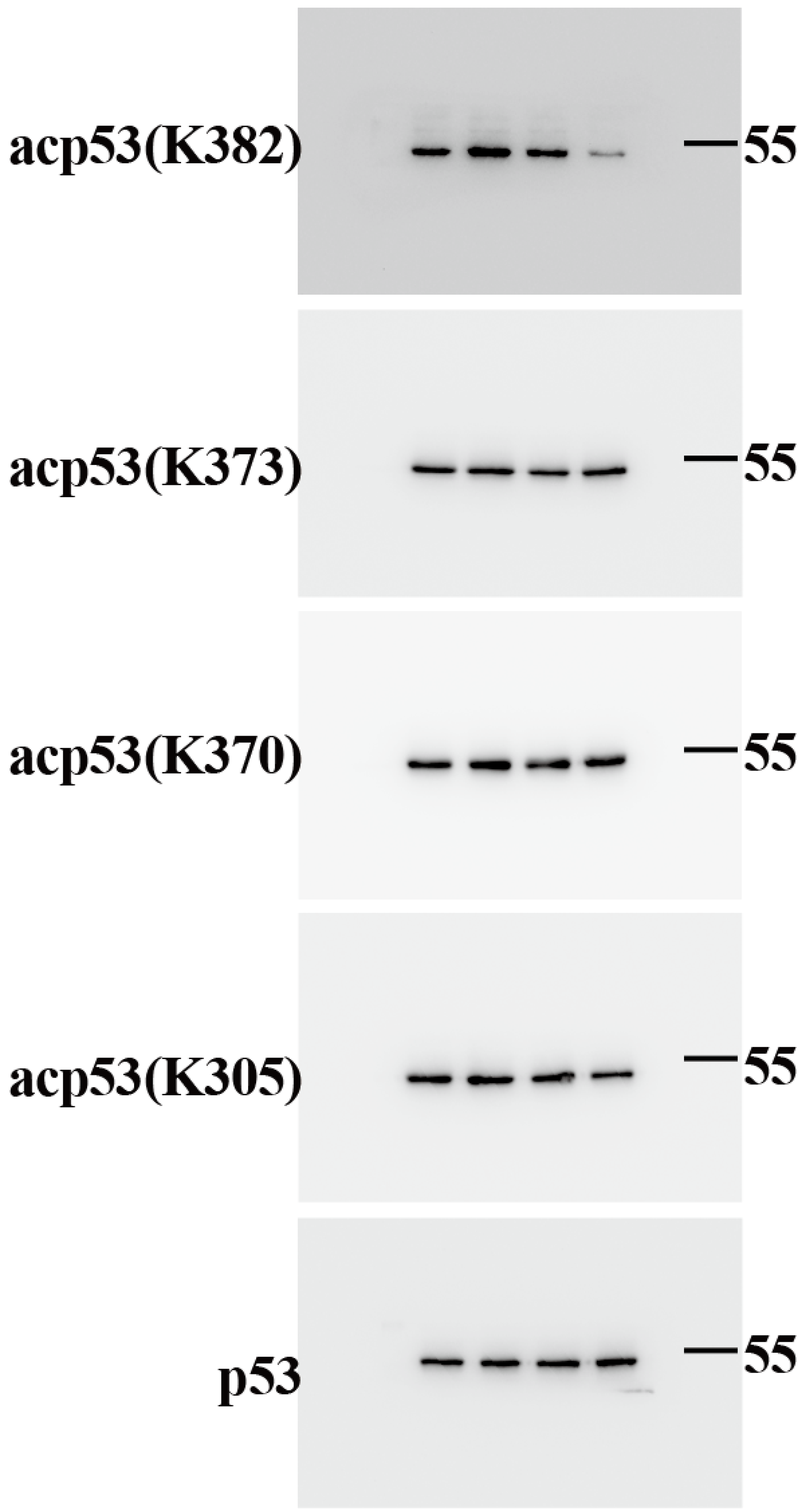

Figure 5M

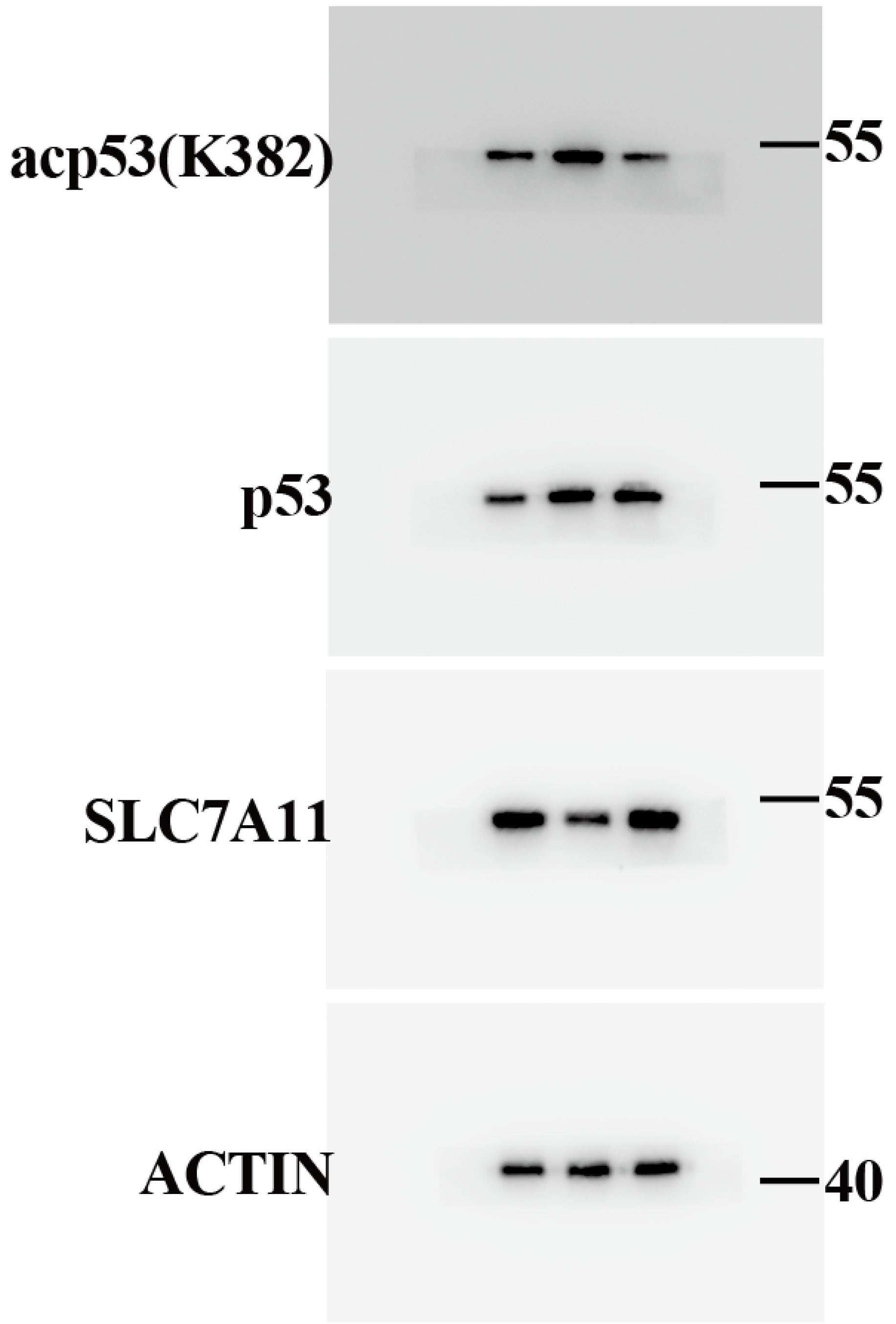

Full and uncropped western blot for Figure 6

Figure 6A

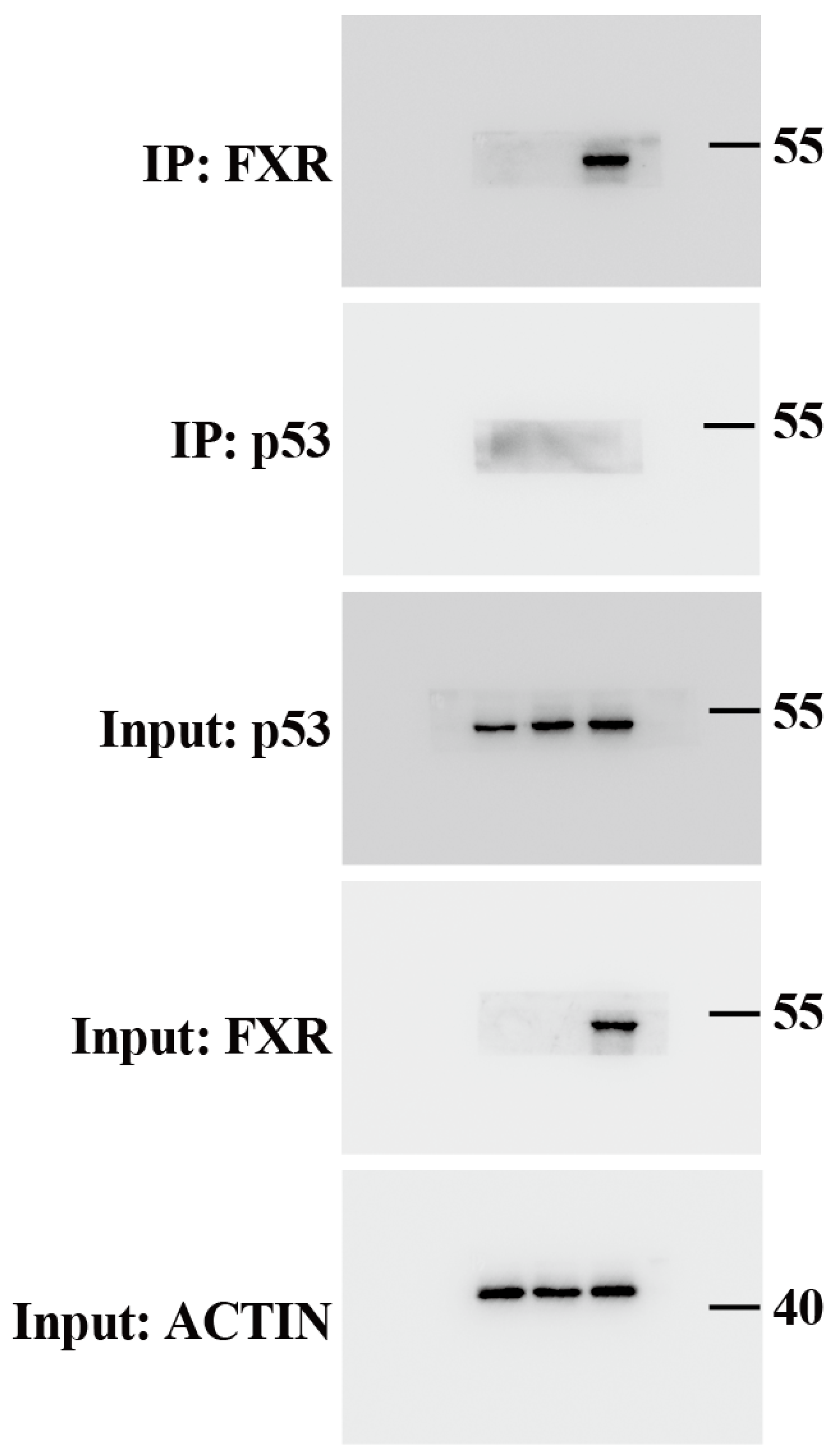

Figure 6C

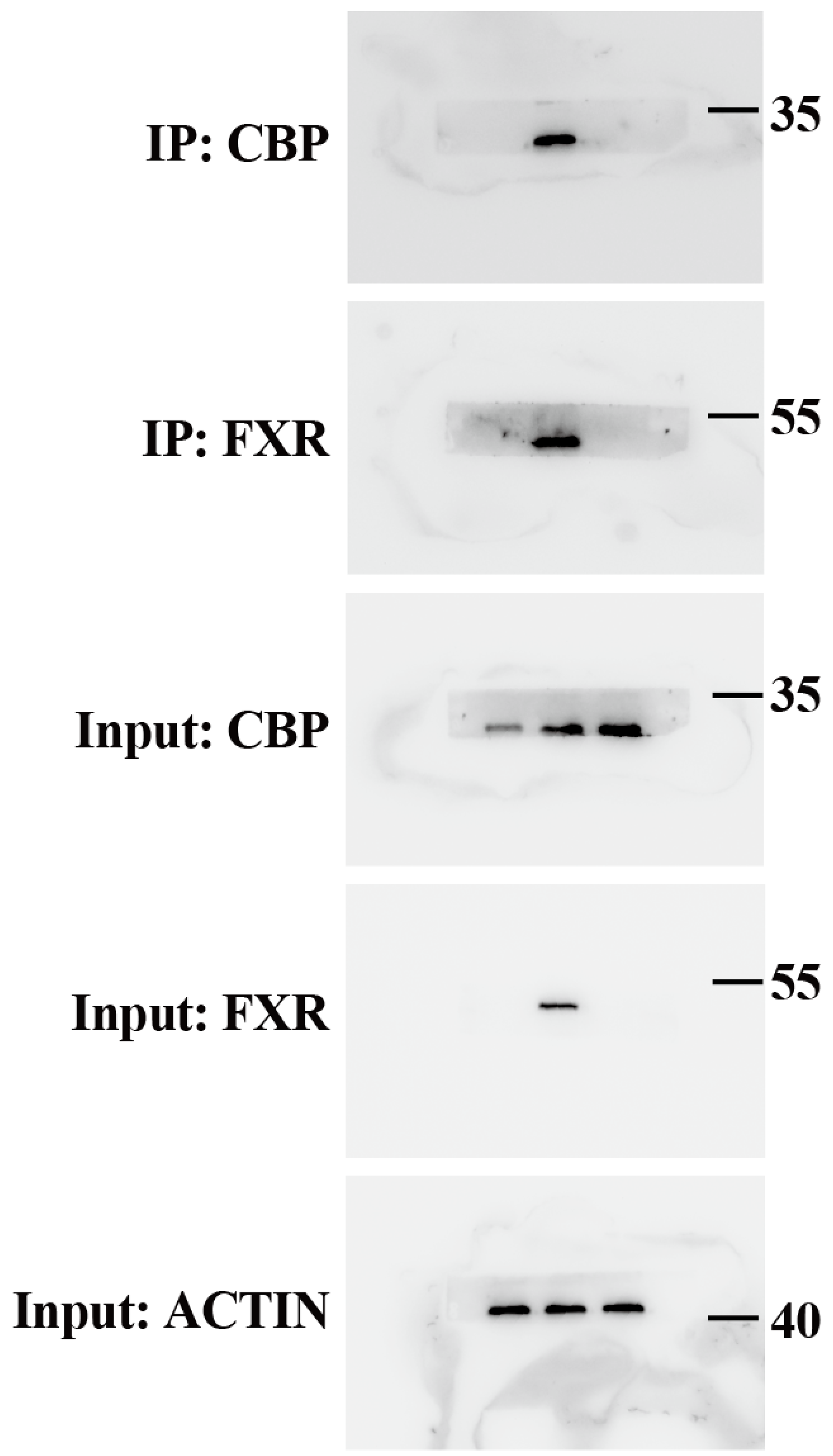

Figure 6D

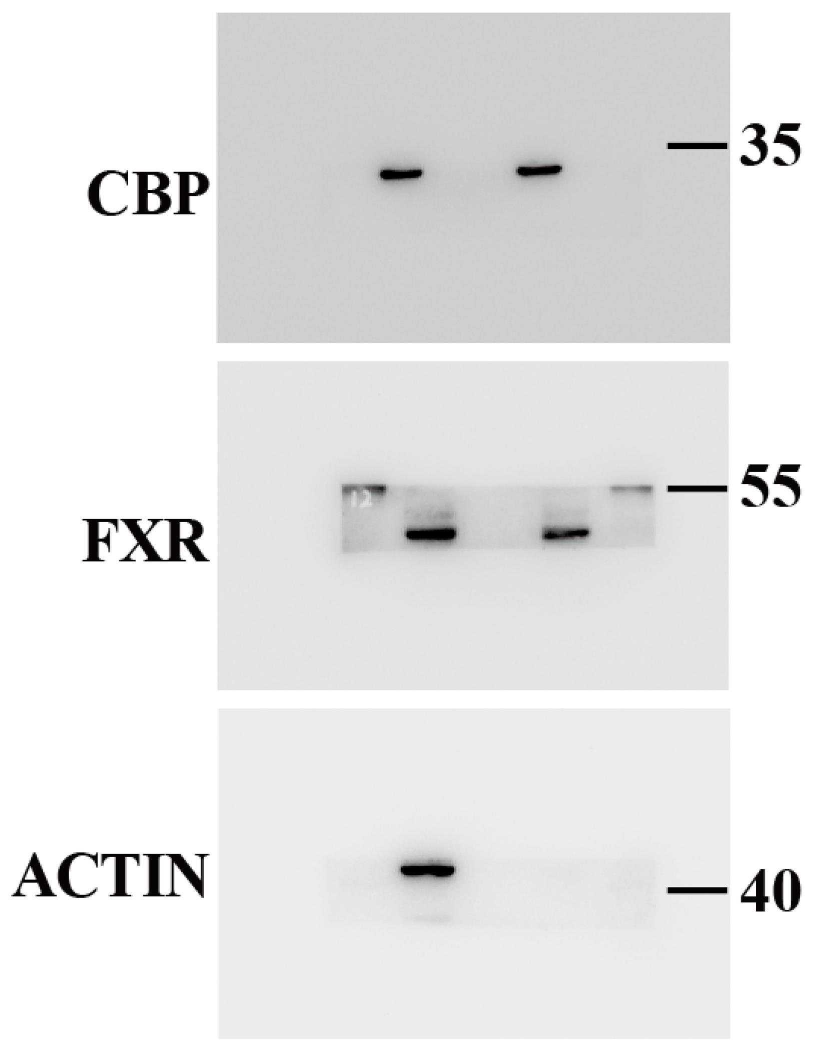

Figure 6E

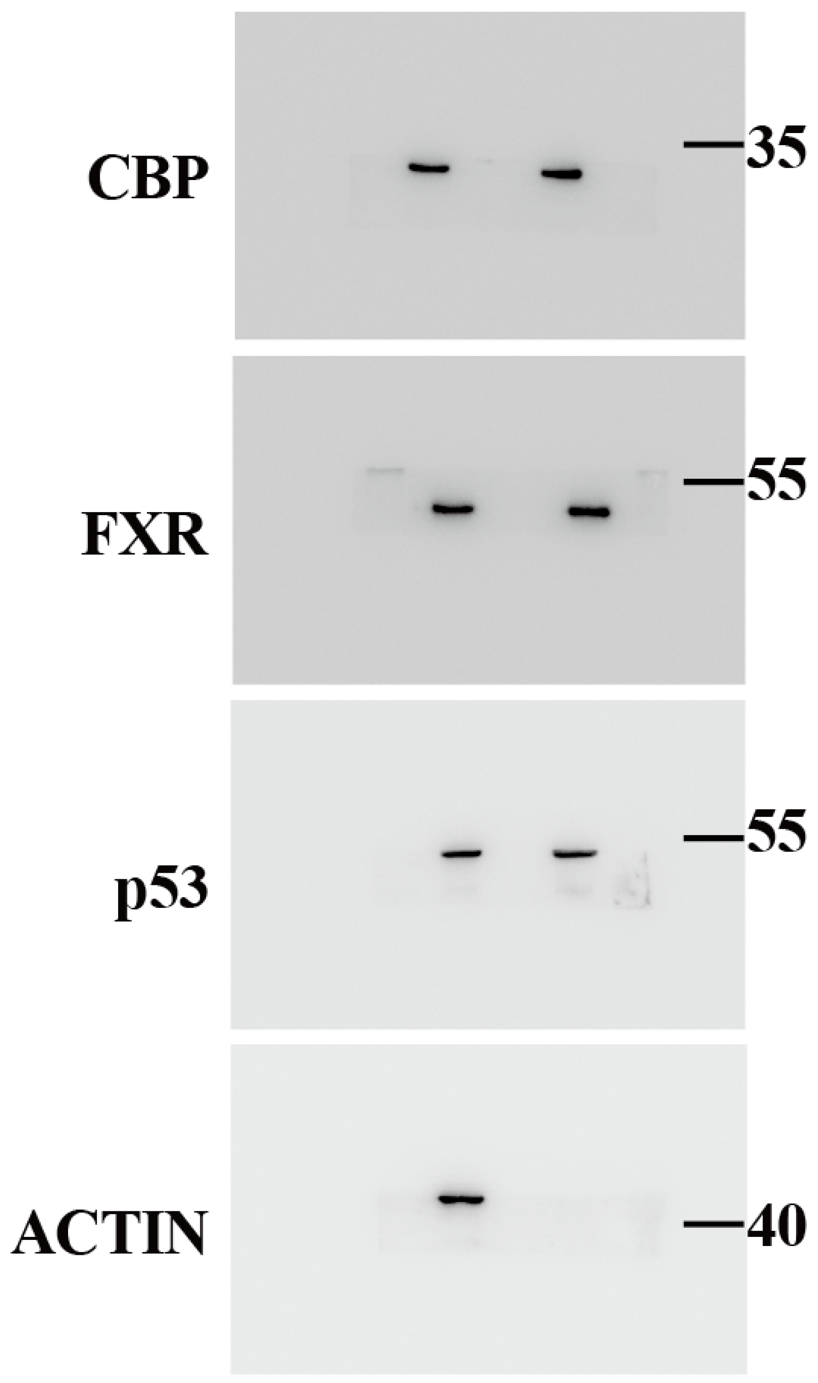

Figure 6H

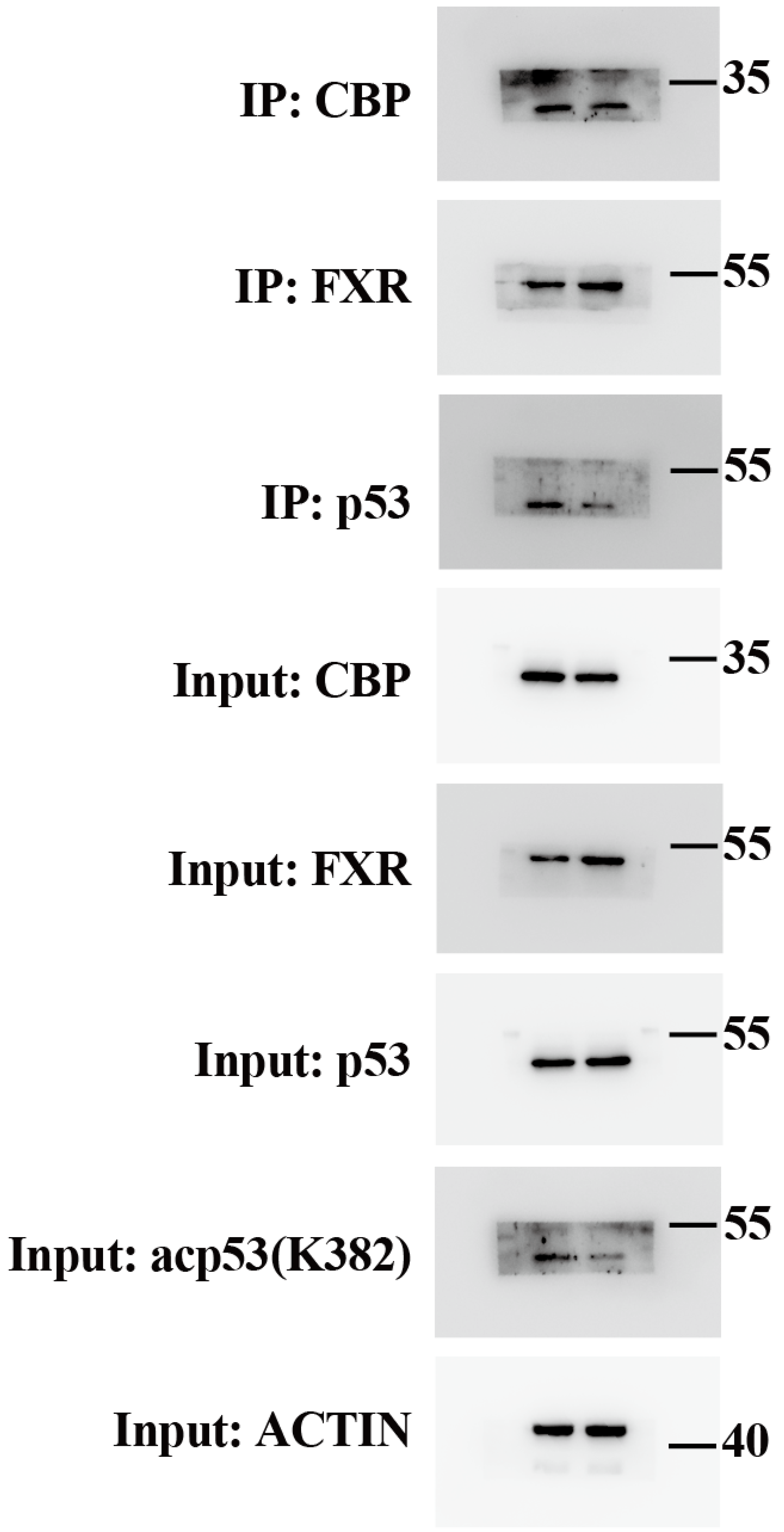

Figure 6K

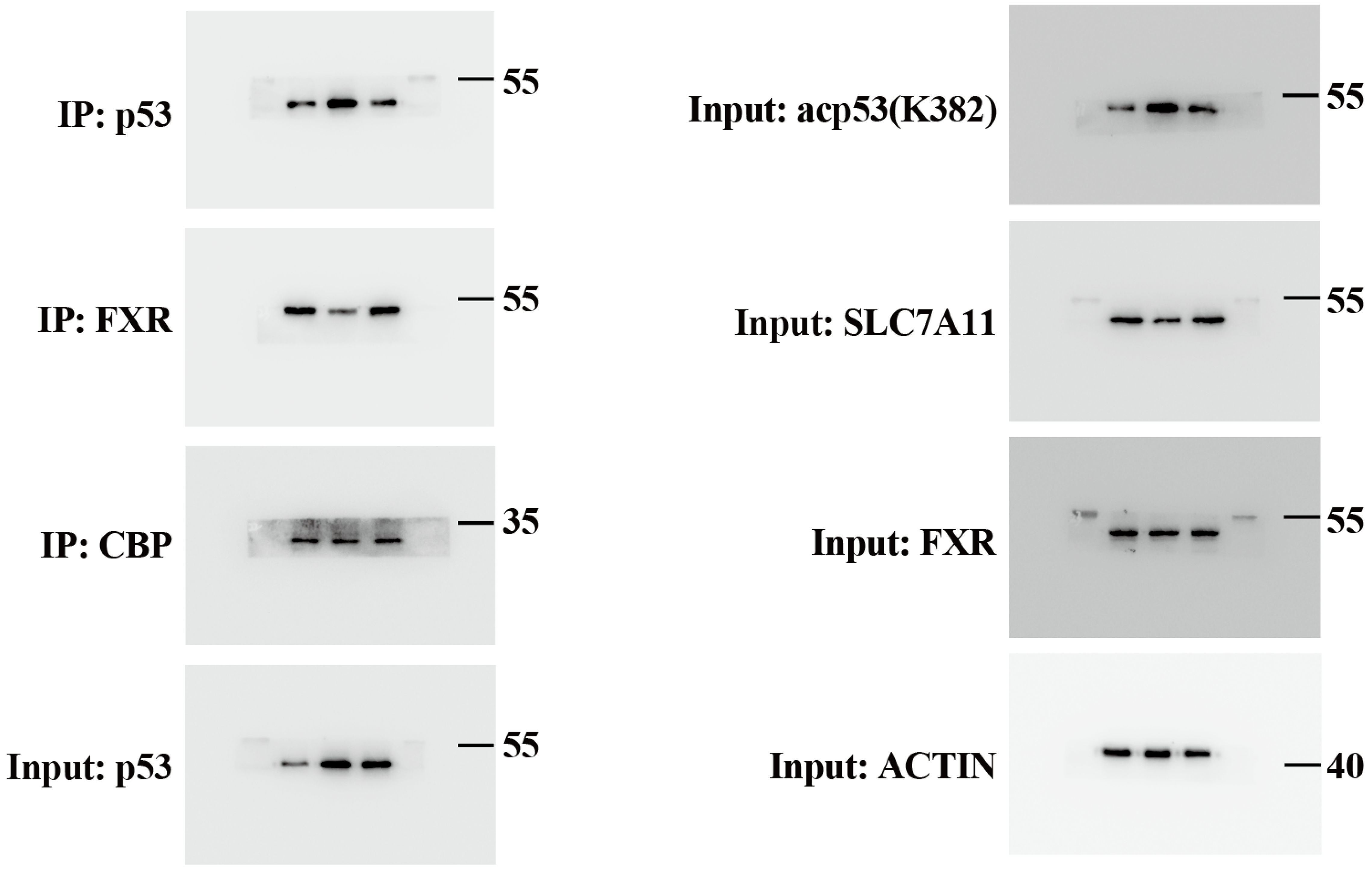

Full and uncropped western blot for Figure 7

Figure 7H

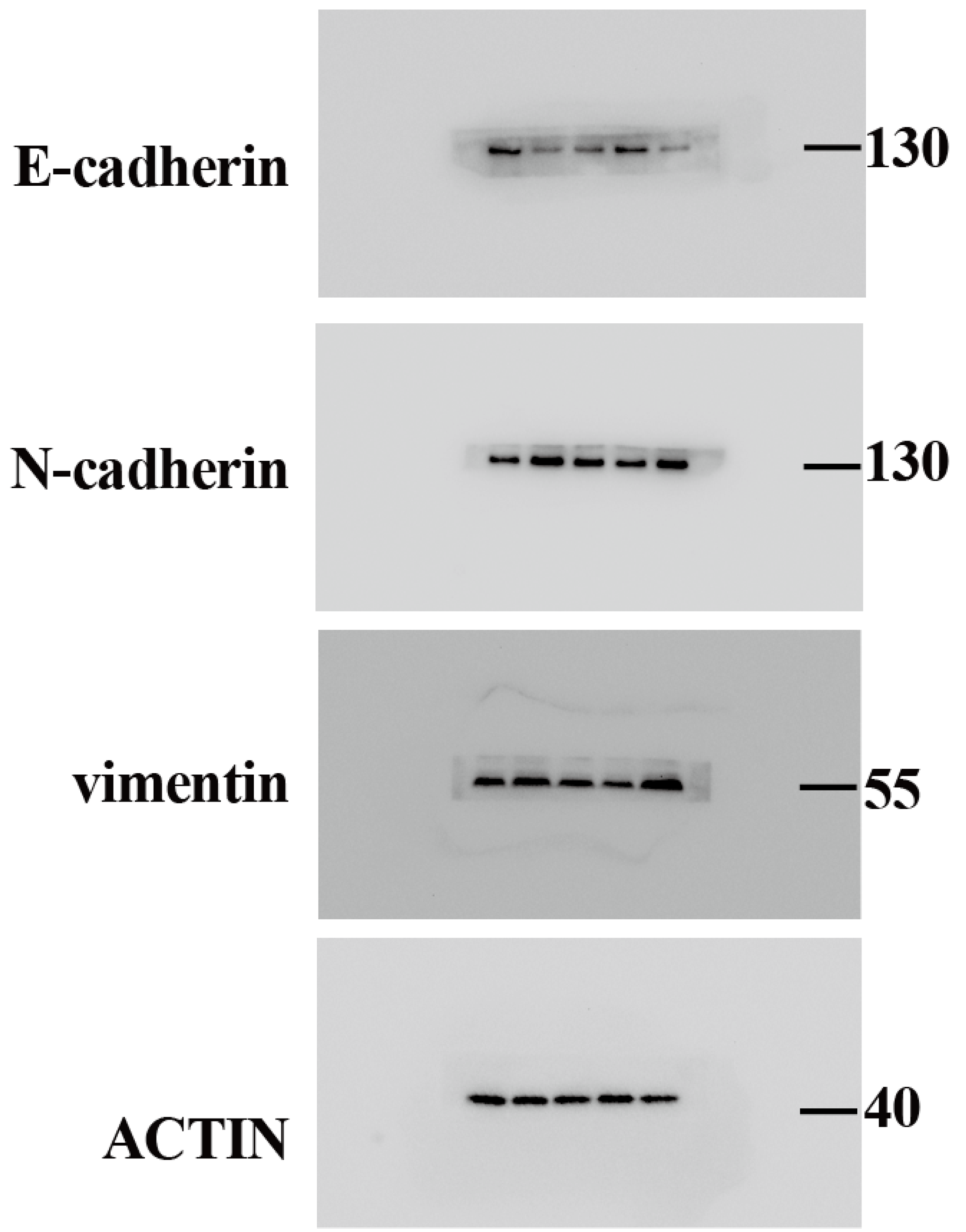

Full and uncropped western blot for Figure 8

Figure 8H

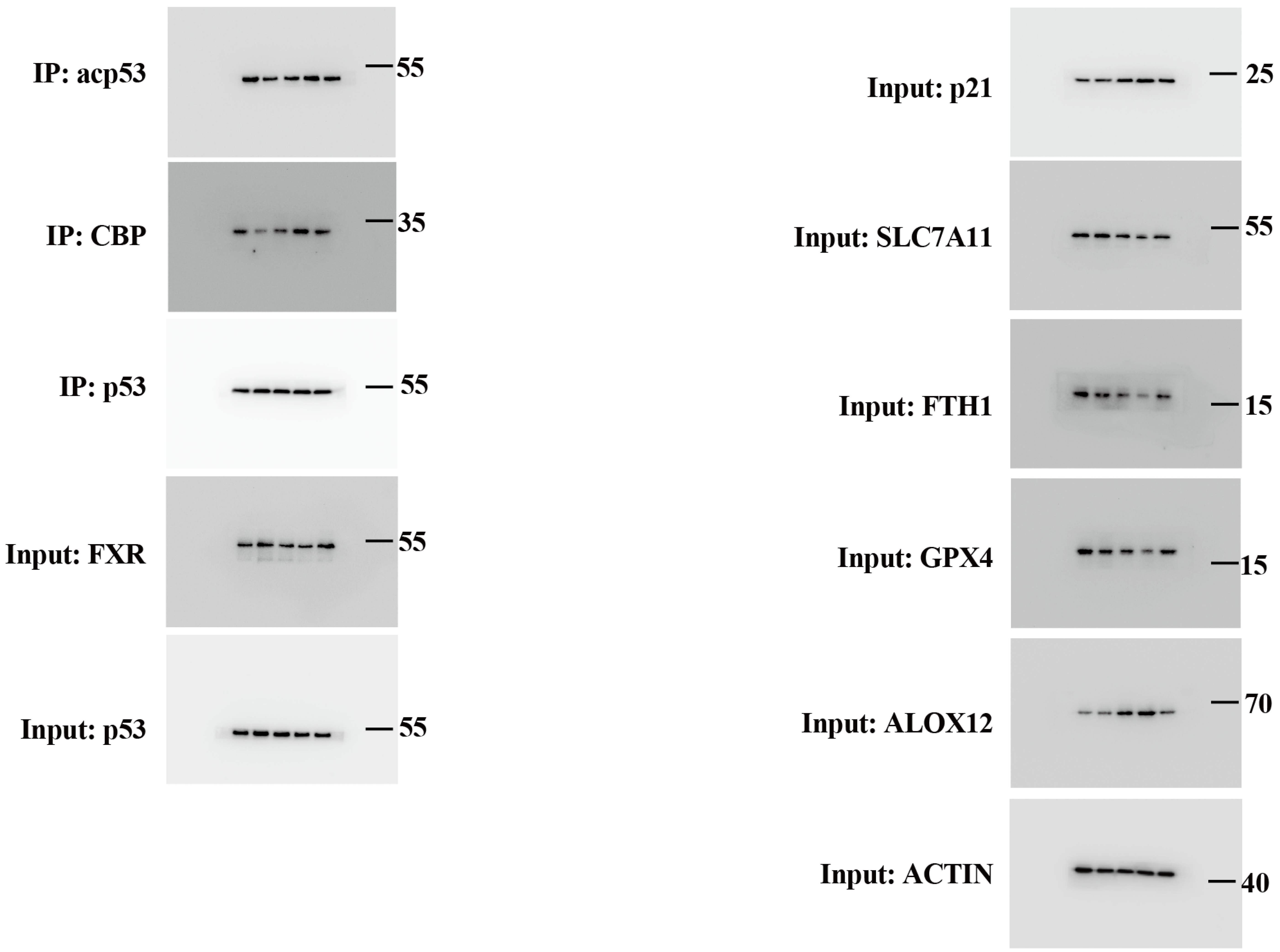

Full and uncropped western blot for Supplementary Figure 2

Figure S2B

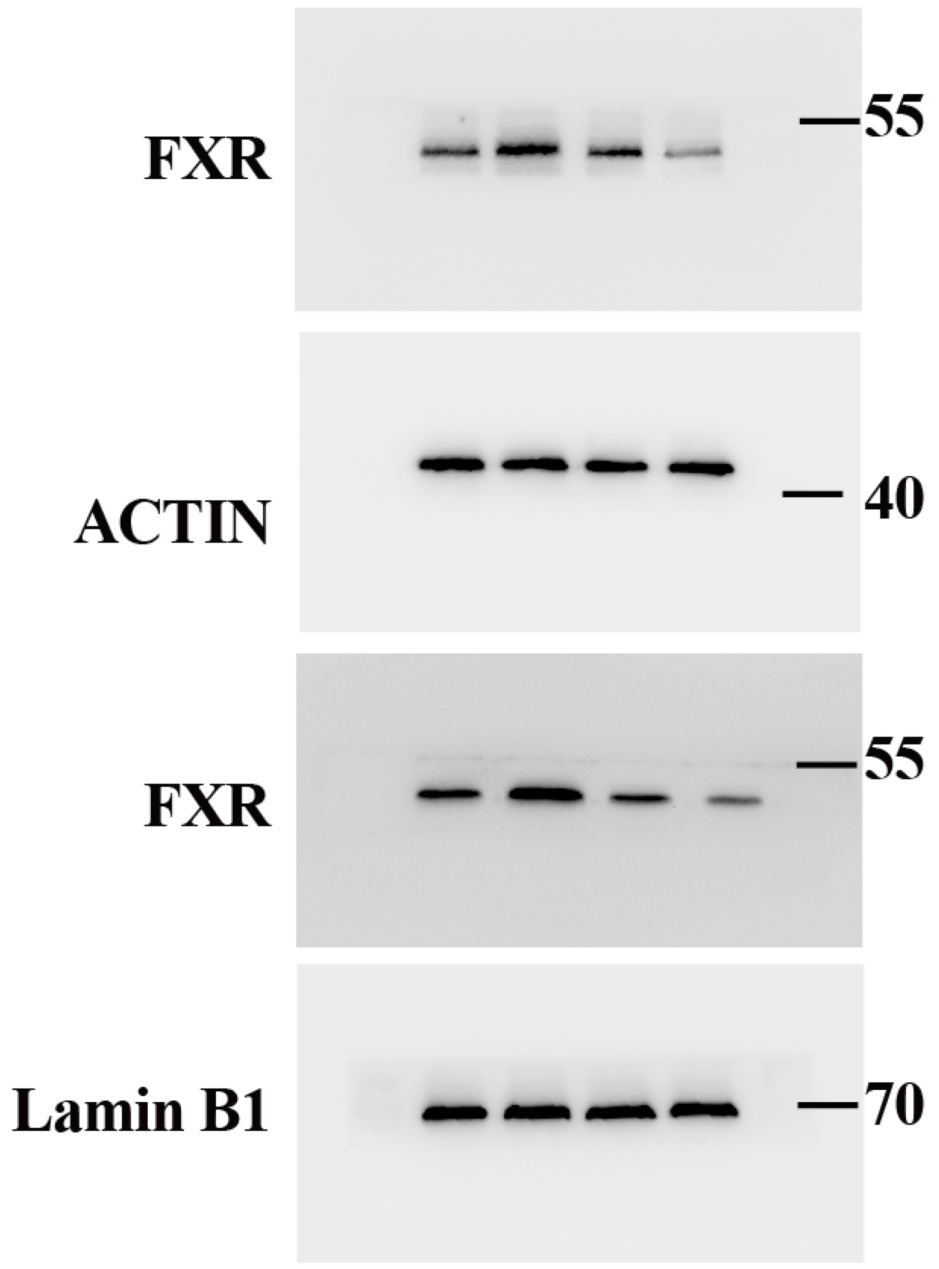

Figure S2E

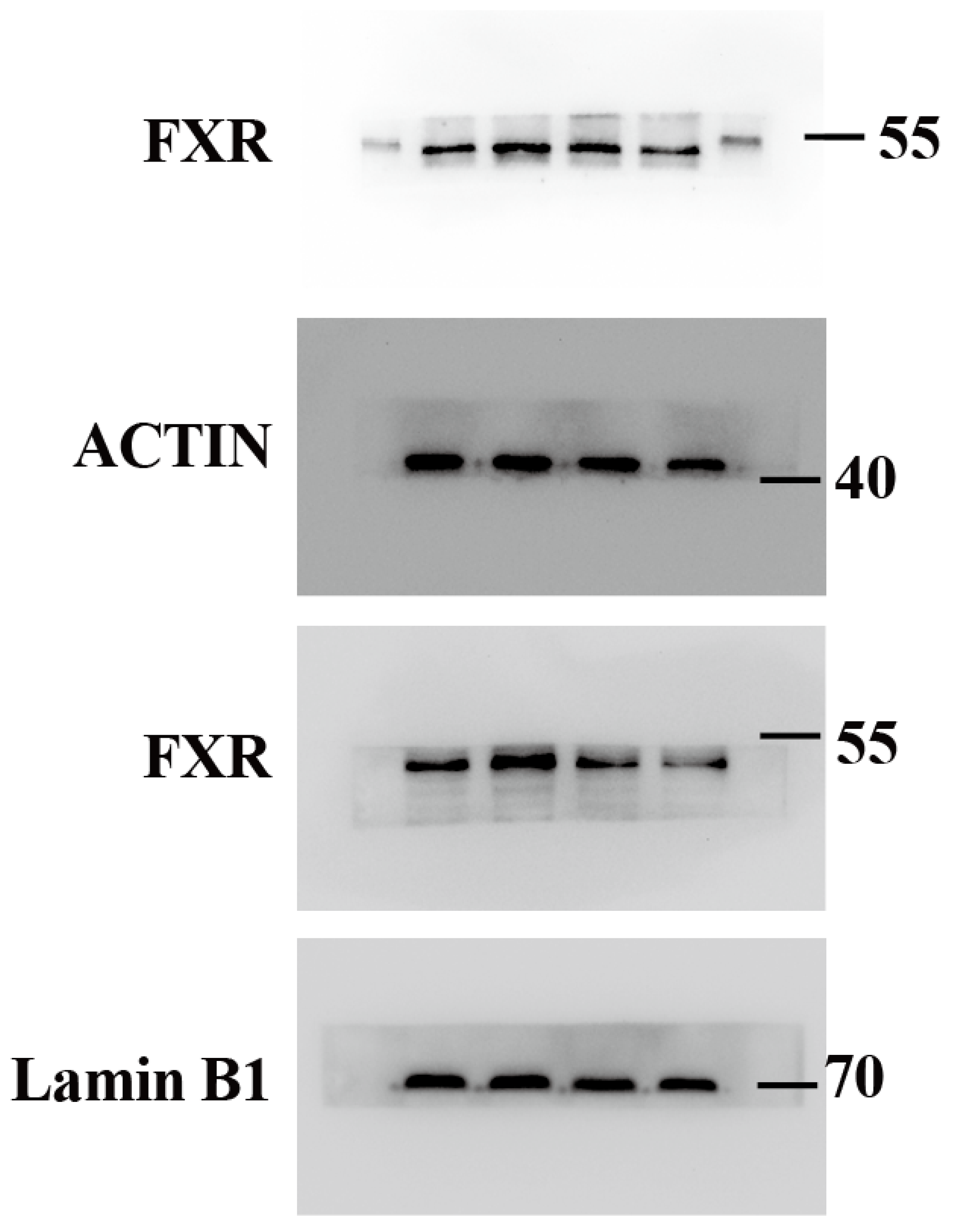

Full and uncropped western blot for Supplementary Figure 3

Figure S3B

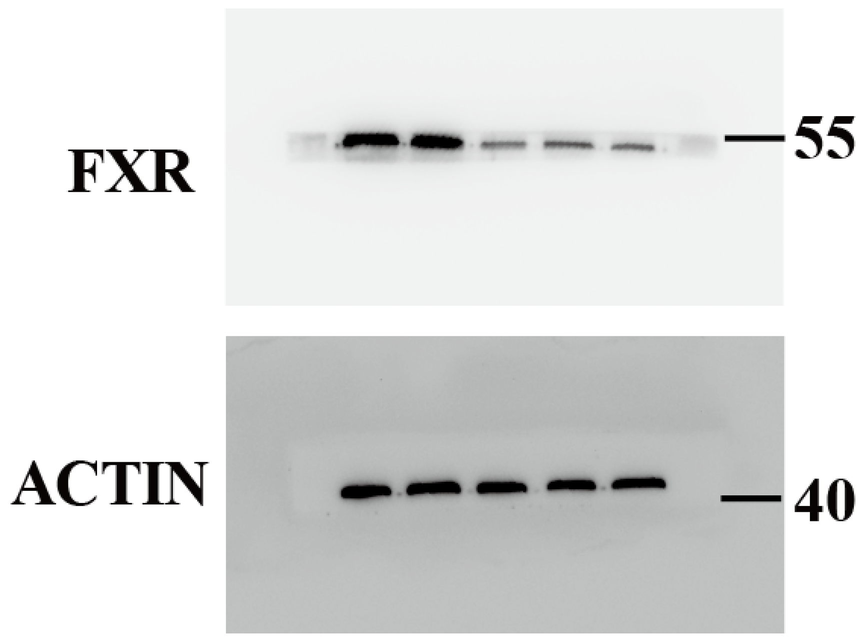

Figure S3D

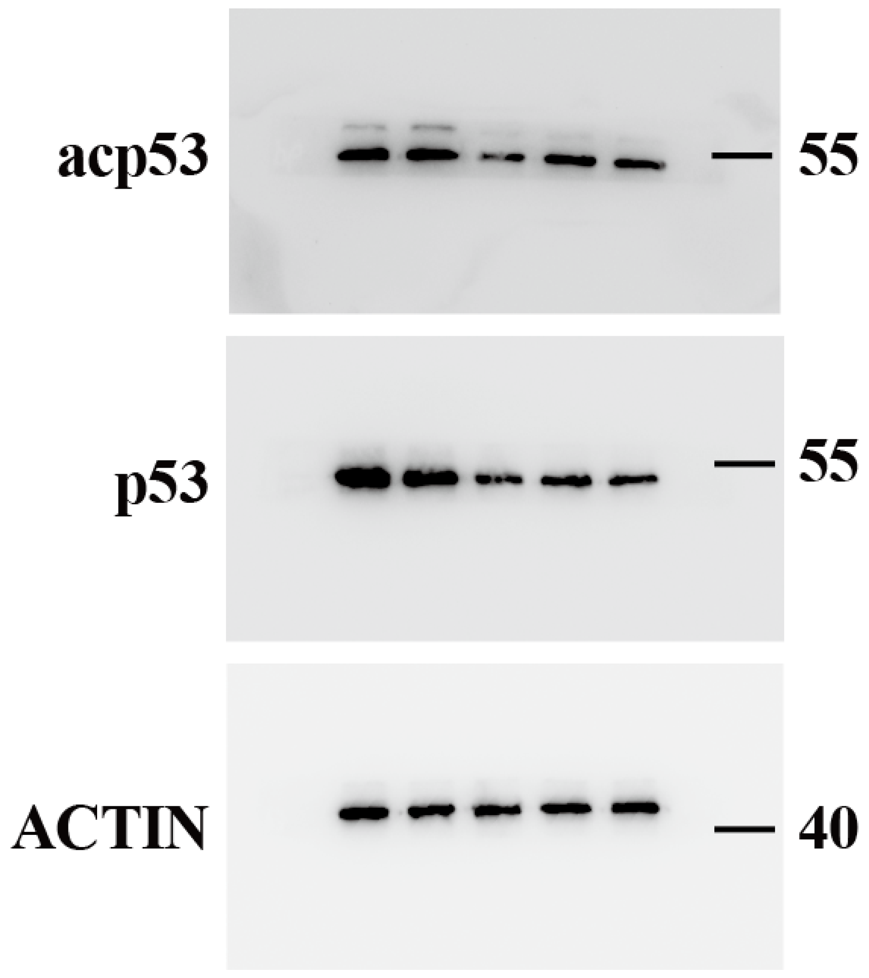

Figure S3F

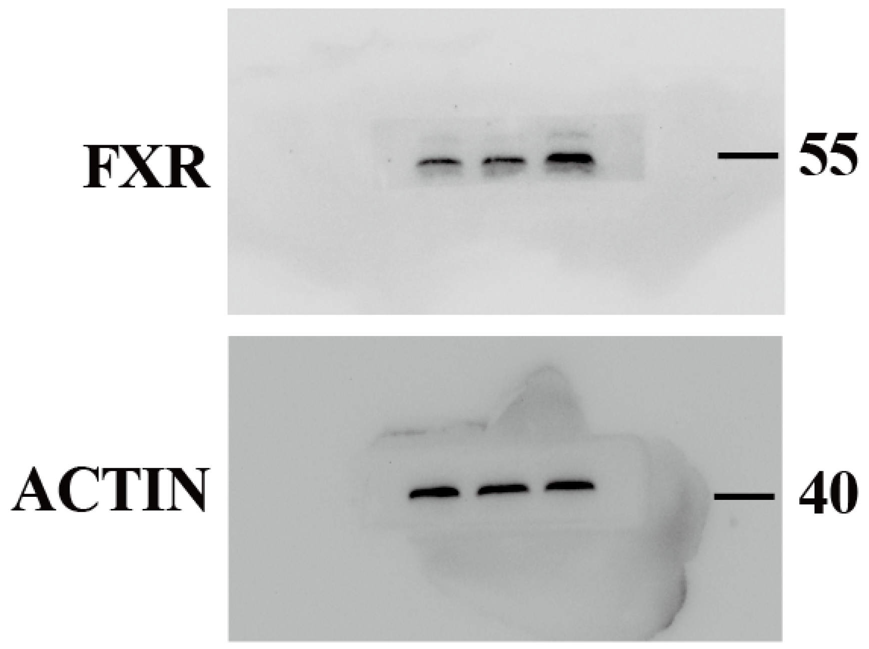

Full and uncropped western blot for Supplementary Figure 5

Figure S5A

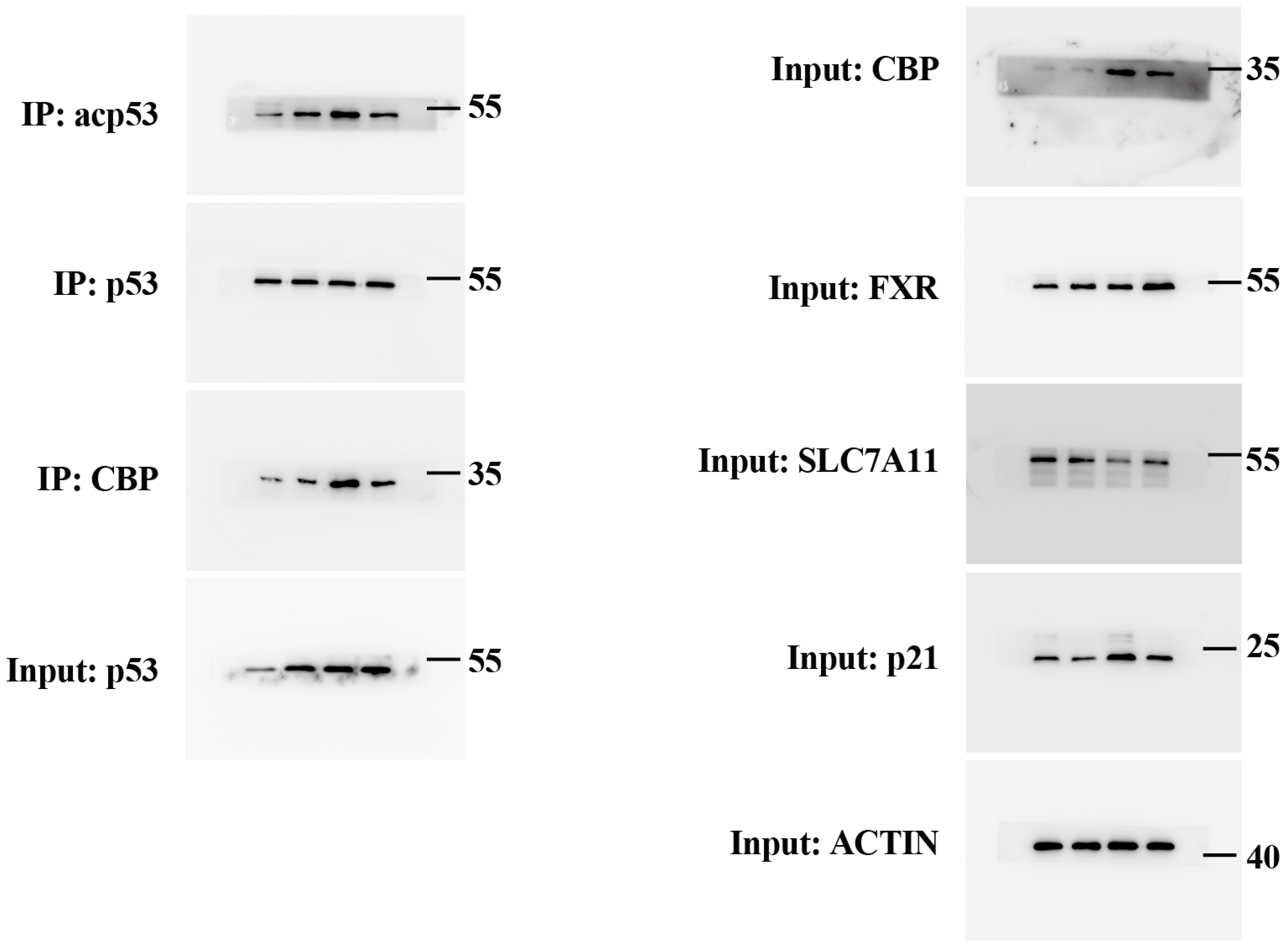

Supplement: Supplementary file 3 — Original Western Blots [file 41419_2024_7222_MOESM3_ESM.pdf]
